# Supplementary material for: Silencing of ANKRD12 circRNA induces molecular and functional changes associated with invasive phenotypes
Source: BMC Cancer. 2019 Jun 11;19:565. doi: 10.1186/s12885-019-5723-0 (PMC6558796; doi:10.1186/s12885-019-5723-0)
Supplement: Supplementary file 1 — Figure S1. (a) The abundance of circANKRD12 and ANKRD12 linear RNA. Semi qRT-PCR data indicating the abundance of circANKRD12 and ANKRD12 mRNA in the cytoplasmic and nuclear fractions of SKOV3 cells, nuclear and cytoplasmic purity markers were also assessed using nuclear specific marker 7SK and cytoplasmic specific marker CYTB. The gel picture represents PCR amplified products of circular RNAs with three different sets of primers. (b) Real-time data shows the abundance of circANKRD12 and ANKRD12 mRNA in the cytoplasm and nucleus. (c) The abundance of circANKRD12 and ANKRD12 mRNA in a panel of cancer cells. (PPTX 2728 kb) (PPTX 2750 kb) [file 12885_2019_5723_MOESM1_ESM.pptx]

## Slide 1
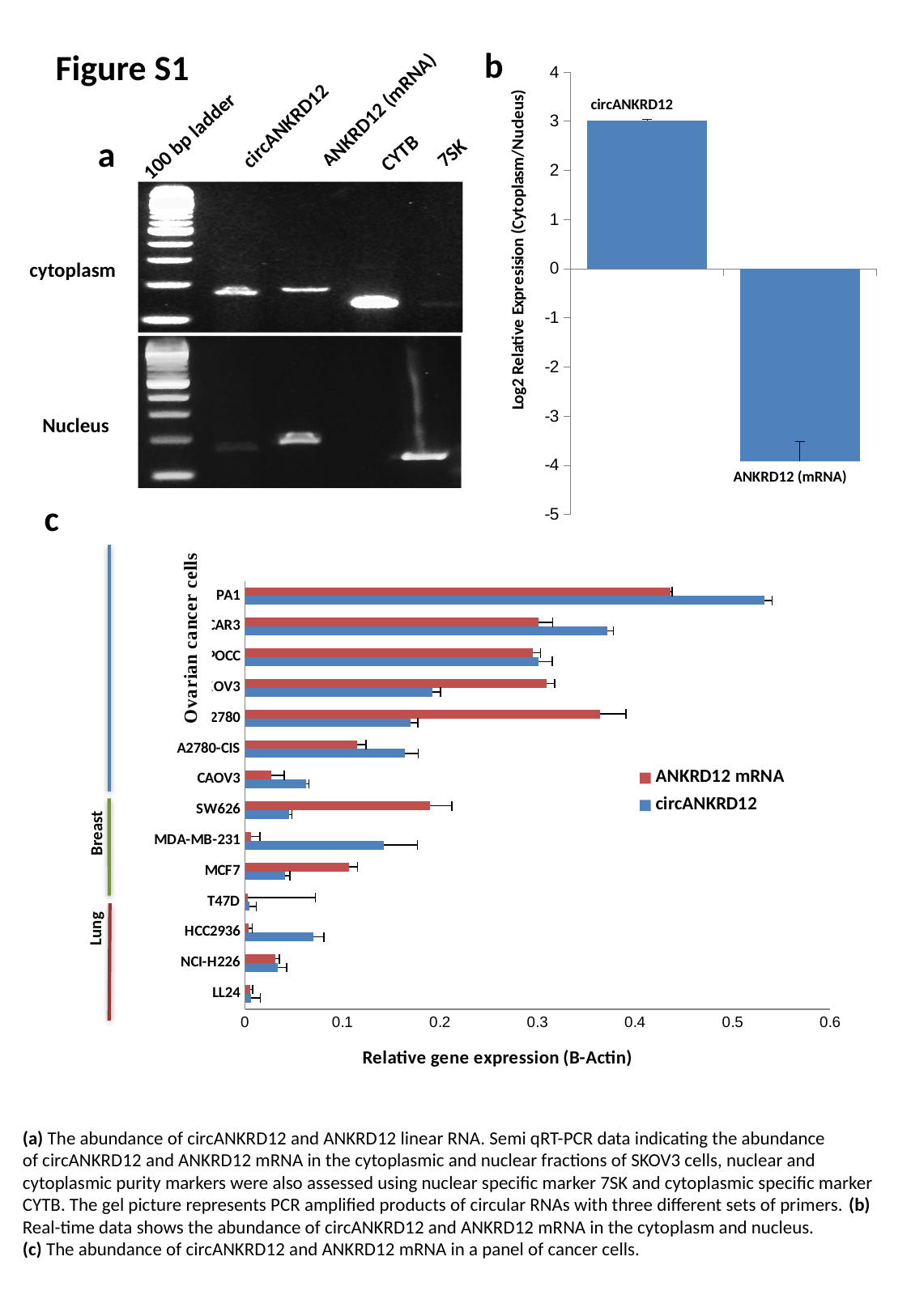

b
Figure S1
100 bp ladder
circANKRD12
a
CYTB
cytoplasm
### Chart
| Category | |
|---|---|
| circANKRD12 | 3.008496641370986 |
| ANKRD12 mRNA | -3.915796097140843 |circANKRD12
ANKRD12 (mRNA)
ANKRD12 (mRNA)
7SK
Nucleus
### Chart
| Category | circANKRD12 | ANKRD12 mRNA |
|---|---|---|
| LL24 | 0.00600210676064188 | 0.00528122614110924 |
| NCI-H226 | 0.0335193198168593 | 0.0309152554771423 |
| HCC2936 | 0.0701078936595648 | 0.00390299781376451 |
| T47D | 0.00473366094143317 | 0.00272586774540201 |
| MCF7 | 0.0413266947112556 | 0.106795393450779 |
| MDA-MB-231 | 0.142377940794671 | 0.00654406109226378 |
| SW626 | 0.0451132052966389 | 0.189378817538427 |
| CAOV3 | 0.0624068426725184 | 0.0268657575055361 |
| A2780-CIS | 0.163916520792635 | 0.114792381258799 |
| A2780 | 0.169667631175748 | 0.364372895017635 |
| SKOV3 | 0.192451420239446 | 0.308943217037822 |
| APOCC | 0.301212898010033 | 0.294907280424192 |
| OVCAR3 | 0.371131795600006 | 0.300871662830001 |
| PA1 | 0.53249035014769 | 0.436073197665764 |c
Breast
Lung
(a) The abundance of circANKRD12 and ANKRD12 linear RNA. Semi qRT-PCR data indicating the abundance
of circANKRD12 and ANKRD12 mRNA in the cytoplasmic and nuclear fractions of SKOV3 cells, nuclear and
cytoplasmic purity markers were also assessed using nuclear specific marker 7SK and cytoplasmic specific marker CYTB. The gel picture represents PCR amplified products of circular RNAs with three different sets of primers. (b)
Real-time data shows the abundance of circANKRD12 and ANKRD12 mRNA in the cytoplasm and nucleus.
(c) The abundance of circANKRD12 and ANKRD12 mRNA in a panel of cancer cells.
