# Supplementary material for: Silencing of ANKRD12 circRNA induces molecular and functional changes associated with invasive phenotypes
Source: BMC Cancer. 2019 Jun 11;19:565. doi: 10.1186/s12885-019-5723-0 (PMC6558796; doi:10.1186/s12885-019-5723-0)
Supplement: Supplementary file 2 — Figure S2. Figure a, b, c Shows functional predicted networks from RNAseq data of circANKRD12 silenced MDA-MB-231 cells induces inflammatory immune responses and cancer cell invasion with activation Z score and p values. (PPTX 6928 kb) (PPTX 6949 kb) [file 12885_2019_5723_MOESM2_ESM.pptx]

## Slide 1
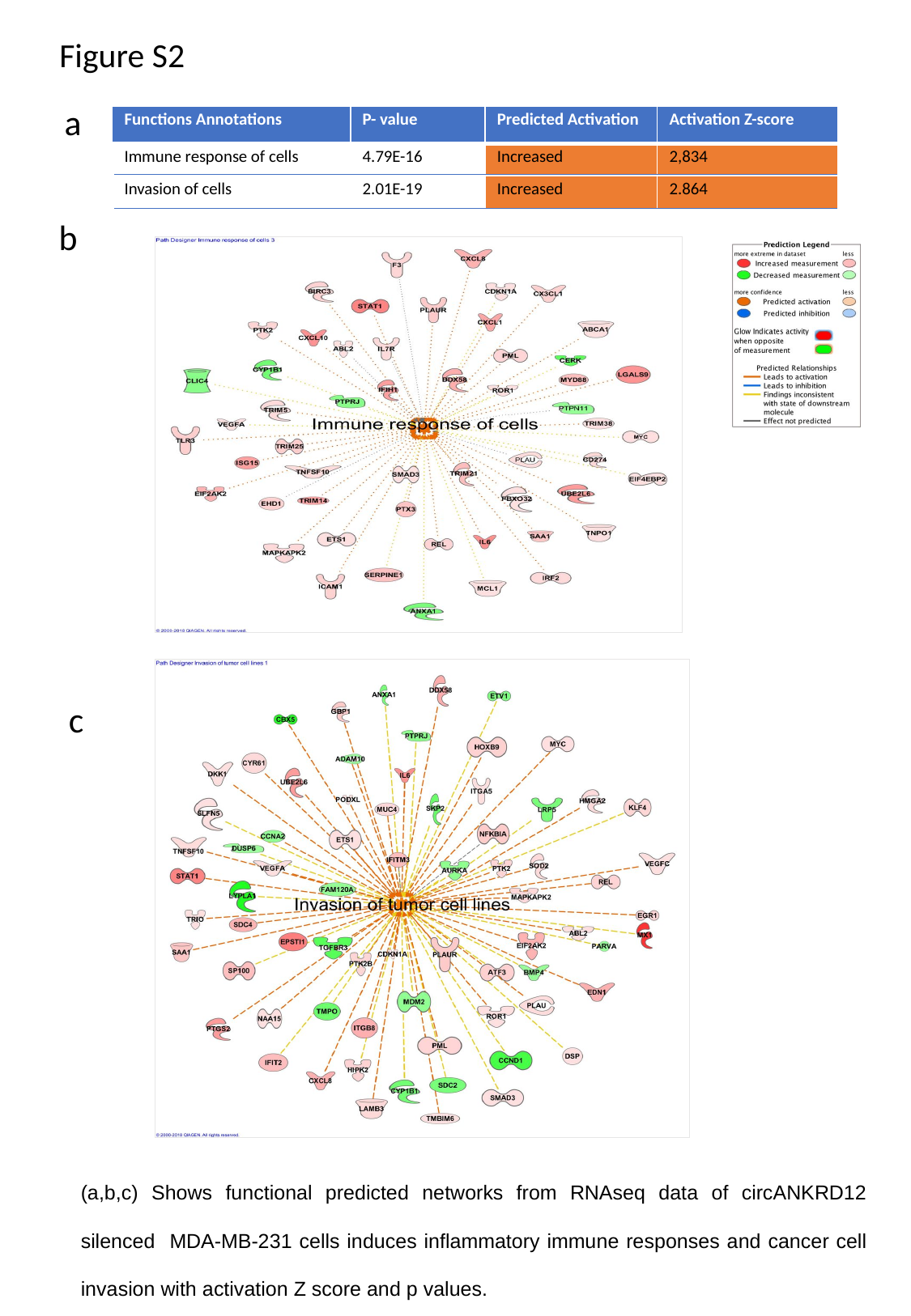

Figure S2
a
| Functions Annotations | P- value | Predicted Activation | Activation Z-score |
| --- | --- | --- | --- |
| Immune response of cells | 4.79E-16 | Increased | 2,834 |
| Invasion of cells | 2.01E-19 | Increased | 2.864 |
b
c
(a,b,c) Shows functional predicted networks from RNAseq data of circANKRD12 silenced MDA-MB-231 cells induces inflammatory immune responses and cancer cell invasion with activation Z score and p values.
