# Supplementary material for: Silencing of ANKRD12 circRNA induces molecular and functional changes associated with invasive phenotypes
Source: BMC Cancer. 2019 Jun 11;19:565. doi: 10.1186/s12885-019-5723-0 (PMC6558796; doi:10.1186/s12885-019-5723-0)
Supplement: Supplementary file 3 — S2. File represents additional figures for validating the circANKRD12 in cell lines. (PPTX 17563 kb) (PPTX 17572 kb) [file 12885_2019_5723_MOESM3_ESM.pptx]

## Slide 1
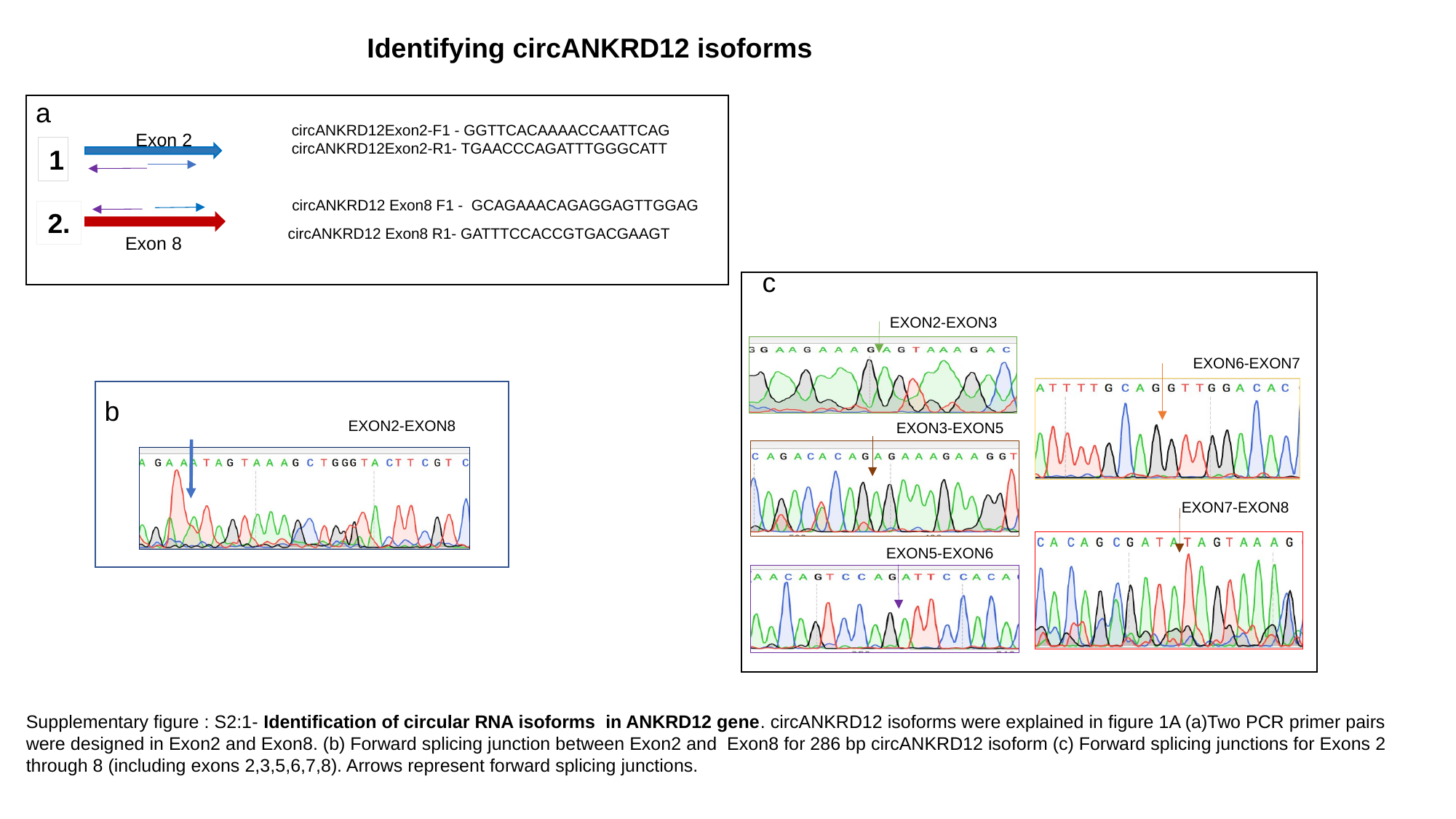

Identifying circANKRD12 isoforms
a
circANKRD12Exon2-F1 - GGTTCACAAAACCAATTCAG
circANKRD12Exon2-R1- TGAACCCAGATTTGGGCATT
Exon 2
1
2.
Exon 8
| circANKRD12 Exon8 F1 - GCAGAAACAGAGGAGTTGGAG |
| --- |
| circANKRD12 Exon8 R1- GATTTCCACCGTGACGAAGT |
c
EXON2-EXON3
EXON6-EXON7
b
EXON2-EXON8
EXON3-EXON5
EXON7-EXON8
EXON5-EXON6
Supplementary figure : S2:1- Identification of circular RNA isoforms in ANKRD12 gene. circANKRD12 isoforms were explained in figure 1A (a)Two PCR primer pairs were designed in Exon2 and Exon8. (b) Forward splicing junction between Exon2 and Exon8 for 286 bp circANKRD12 isoform (c) Forward splicing junctions for Exons 2 through 8 (including exons 2,3,5,6,7,8). Arrows represent forward splicing junctions.

## Slide 2
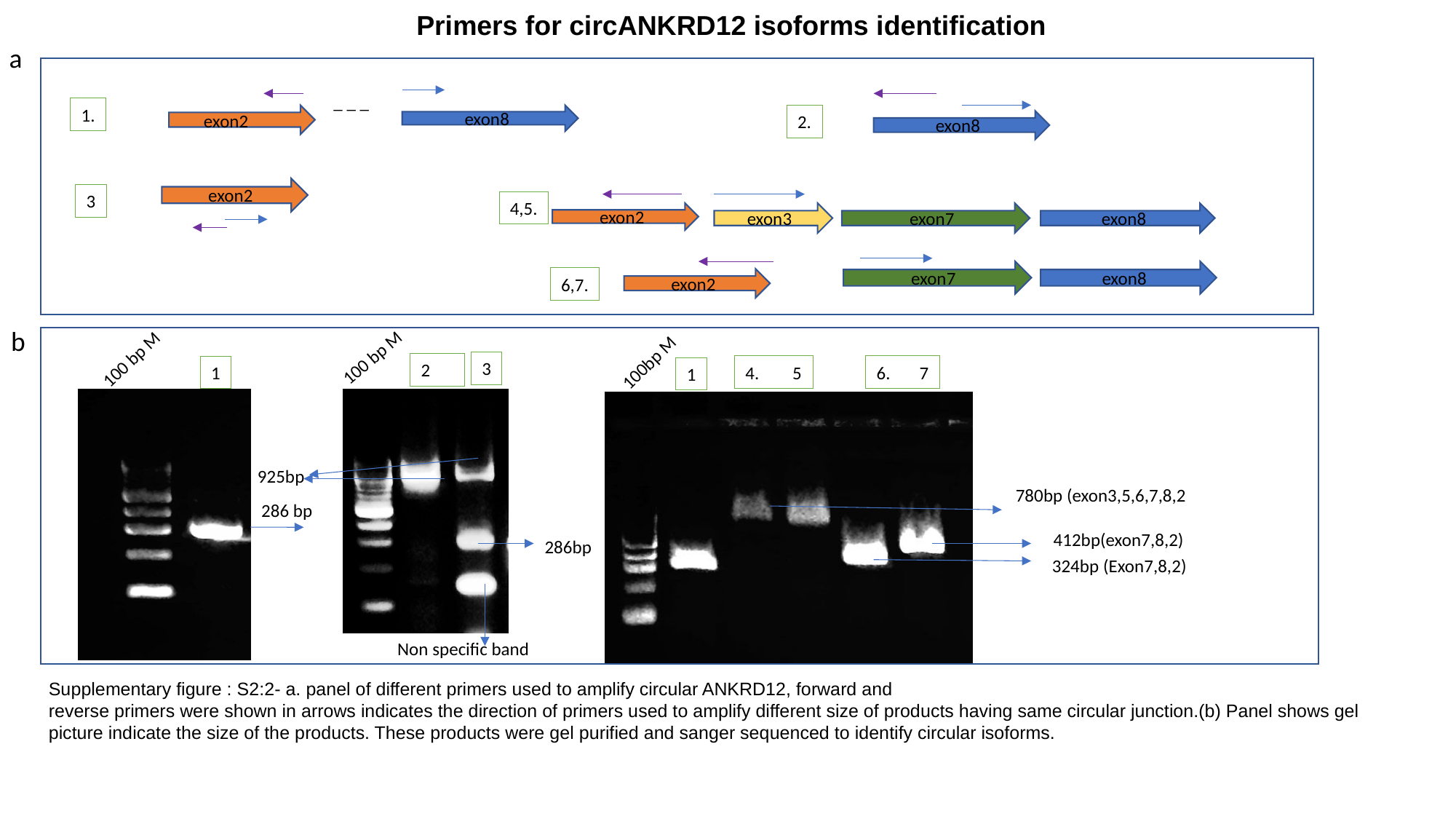

Primers for circANKRD12 isoforms identification
a
_ _ _
1.
exon2
exon8
2.
exon8
exon2
3
4,5.
exon7
exon8
exon2
exon3
exon8
exon7
6,7.
exon2
b
100 bp M
100 bp M
100bp M
3
2
6. 7
4. 5
1
1
925bp
286 bp
412bp(exon7,8,2)
286bp
324bp (Exon7,8,2)
Non specific band
780bp (exon3,5,6,7,8,2
Supplementary figure : S2:2- a. panel of different primers used to amplify circular ANKRD12, forward and
reverse primers were shown in arrows indicates the direction of primers used to amplify different size of products having same circular junction.(b) Panel shows gel picture indicate the size of the products. These products were gel purified and sanger sequenced to identify circular isoforms.

## Slide 3
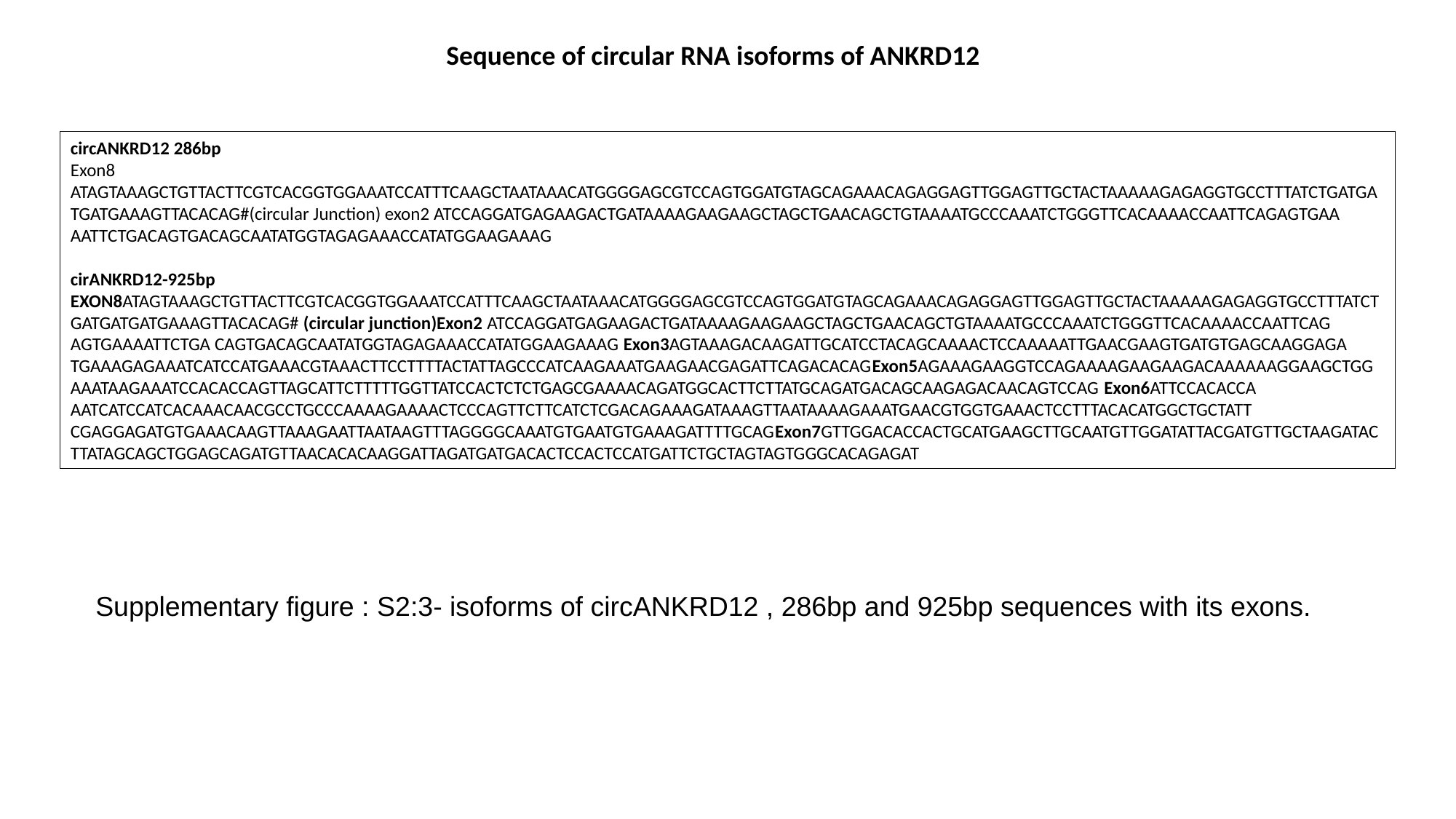

Sequence of circular RNA isoforms of ANKRD12
circANKRD12 286bp
Exon8 ATAGTAAAGCTGTTACTTCGTCACGGTGGAAATCCATTTCAAGCTAATAAACATGGGGAGCGTCCAGTGGATGTAGCAGAAACAGAGGAGTTGGAGTTGCTACTAAAAAGAGAGGTGCCTTTATCTGATGATGATGAAAGTTACACAG#(circular Junction) exon2 ATCCAGGATGAGAAGACTGATAAAAGAAGAAGCTAGCTGAACAGCTGTAAAATGCCCAAATCTGGGTTCACAAAACCAATTCAGAGTGAA
AATTCTGACAGTGACAGCAATATGGTAGAGAAACCATATGGAAGAAAG
cirANKRD12-925bp
EXON8ATAGTAAAGCTGTTACTTCGTCACGGTGGAAATCCATTTCAAGCTAATAAACATGGGGAGCGTCCAGTGGATGTAGCAGAAACAGAGGAGTTGGAGTTGCTACTAAAAAGAGAGGTGCCTTTATCTGATGATGATGAAAGTTACACAG# (circular junction)Exon2 ATCCAGGATGAGAAGACTGATAAAAGAAGAAGCTAGCTGAACAGCTGTAAAATGCCCAAATCTGGGTTCACAAAACCAATTCAG
AGTGAAAATTCTGA CAGTGACAGCAATATGGTAGAGAAACCATATGGAAGAAAG Exon3AGTAAAGACAAGATTGCATCCTACAGCAAAACTCCAAAAATTGAACGAAGTGATGTGAGCAAGGAGA
TGAAAGAGAAATCATCCATGAAACGTAAACTTCCTTTTACTATTAGCCCATCAAGAAATGAAGAACGAGATTCAGACACAGExon5AGAAAGAAGGTCCAGAAAAGAAGAAGACAAAAAAGGAAGCTGGAAATAAGAAATCCACACCAGTTAGCATTCTTTTTGGTTATCCACTCTCTGAGCGAAAACAGATGGCACTTCTTATGCAGATGACAGCAAGAGACAACAGTCCAG Exon6ATTCCACACCA
AATCATCCATCACAAACAACGCCTGCCCAAAAGAAAACTCCCAGTTCTTCATCTCGACAGAAAGATAAAGTTAATAAAAGAAATGAACGTGGTGAAACTCCTTTACACATGGCTGCTATT
CGAGGAGATGTGAAACAAGTTAAAGAATTAATAAGTTTAGGGGCAAATGTGAATGTGAAAGATTTTGCAGExon7GTTGGACACCACTGCATGAAGCTTGCAATGTTGGATATTACGATGTTGCTAAGATACTTATAGCAGCTGGAGCAGATGTTAACACACAAGGATTAGATGATGACACTCCACTCCATGATTCTGCTAGTAGTGGGCACAGAGAT
Supplementary figure : S2:3- isoforms of circANKRD12 , 286bp and 925bp sequences with its exons.

## Slide 4
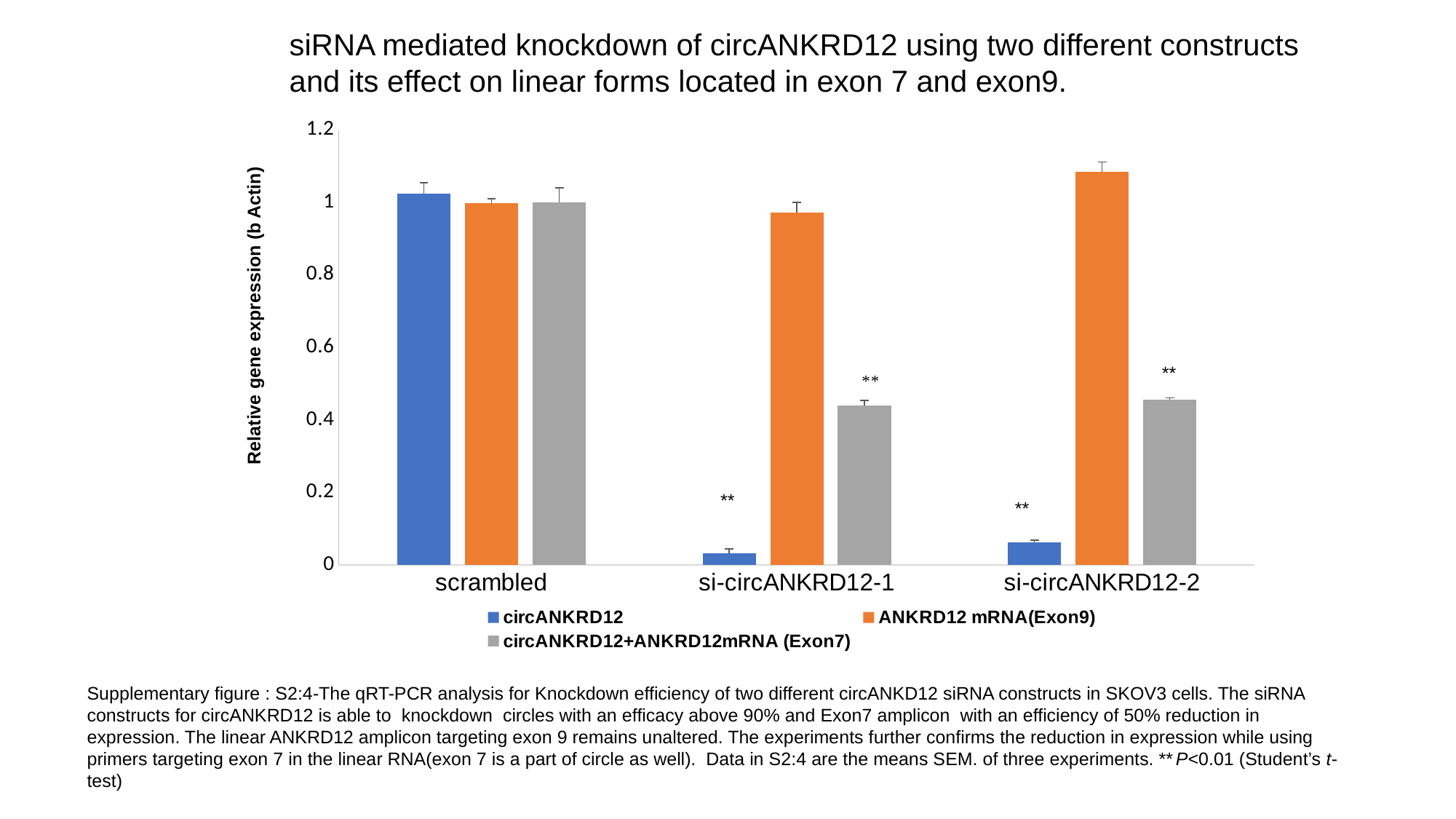

siRNA mediated knockdown of circANKRD12 using two different constructs and its effect on linear forms located in exon 7 and exon9.
### Chart
| Category | circANKRD12 | ANKRD12 mRNA(Exon9) | circANKRD12+ANKRD12mRNA (Exon7) |
|---|---|---|---|
| scrambled | 1.02507579698437 | 0.997936054957155 | 1.000802999971083 |
| si-circANKRD12-1 | 0.0334968300790866 | 0.97139418636 | 0.440880239777751 |
| si-circANKRD12-2 | 0.0621214102993031 | 1.084049108024392 | 0.456266346162016 |Relative gene expression (b Actin)
**
 **
**
Supplementary figure : S2:4-The qRT-PCR analysis for Knockdown efficiency of two different circANKD12 siRNA constructs in SKOV3 cells. The siRNA constructs for circANKRD12 is able to knockdown circles with an efficacy above 90% and Exon7 amplicon with an efficiency of 50% reduction in expression. The linear ANKRD12 amplicon targeting exon 9 remains unaltered. The experiments further confirms the reduction in expression while using primers targeting exon 7 in the linear RNA(exon 7 is a part of circle as well). Data in S2:4 are the means SEM. of three experiments. **P<0.01 (Student’s t-test)

## Slide 5
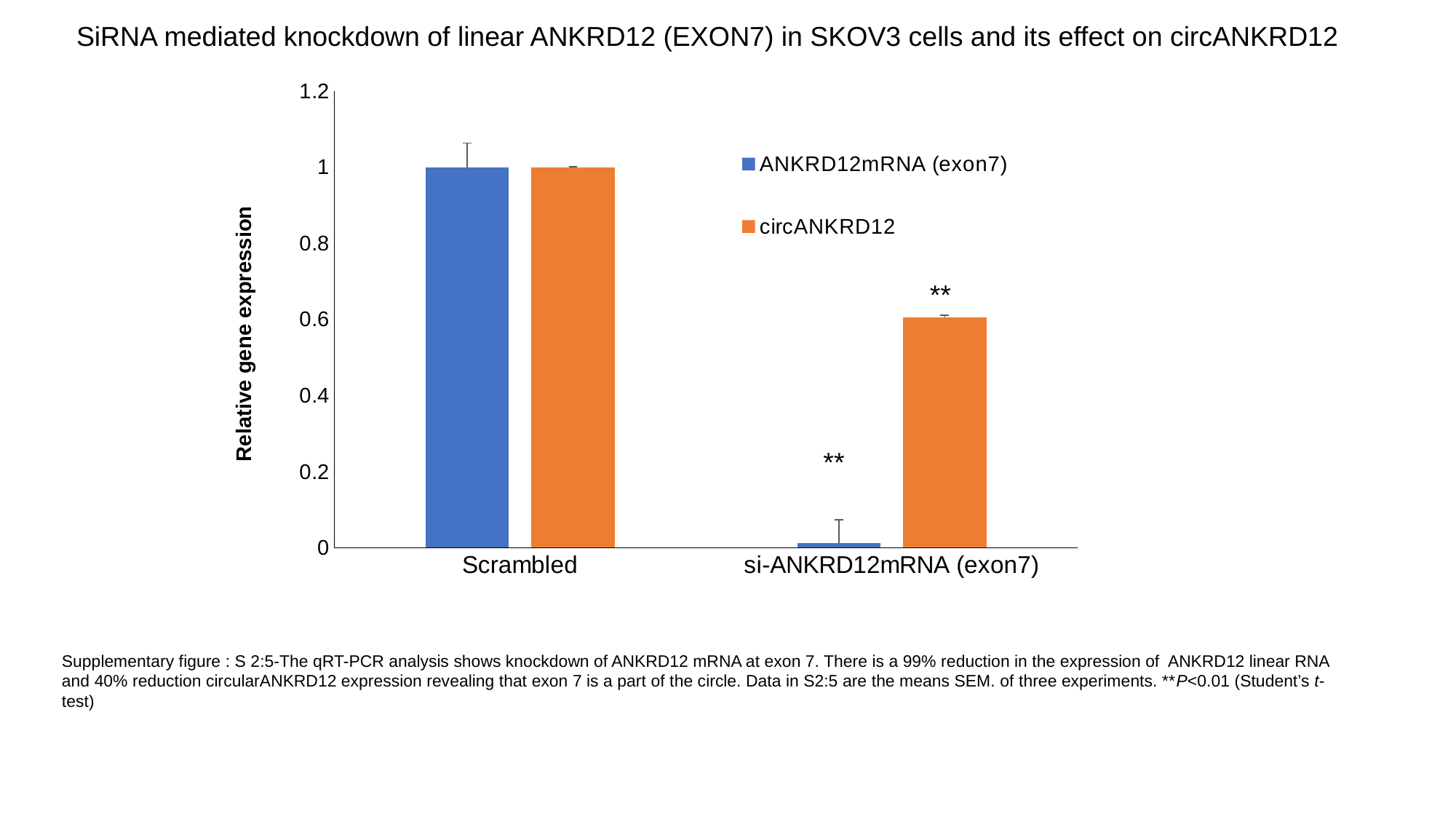

SiRNA mediated knockdown of linear ANKRD12 (EXON7) in SKOV3 cells and its effect on circANKRD12
### Chart
| Category | ANKRD12mRNA (exon7) | circANKRD12 |
|---|---|---|
| Scrambled | 1.0 | 1.0 |
| si-ANKRD12mRNA (exon7) | 0.012496763 | 0.6045763 |
### Chart
| Category |
|---|**
Relative gene expression
**
Supplementary figure : S 2:5-The qRT-PCR analysis shows knockdown of ANKRD12 mRNA at exon 7. There is a 99% reduction in the expression of ANKRD12 linear RNA and 40% reduction circularANKRD12 expression revealing that exon 7 is a part of the circle. Data in S2:5 are the means SEM. of three experiments. **P<0.01 (Student’s t-test)

## Slide 6
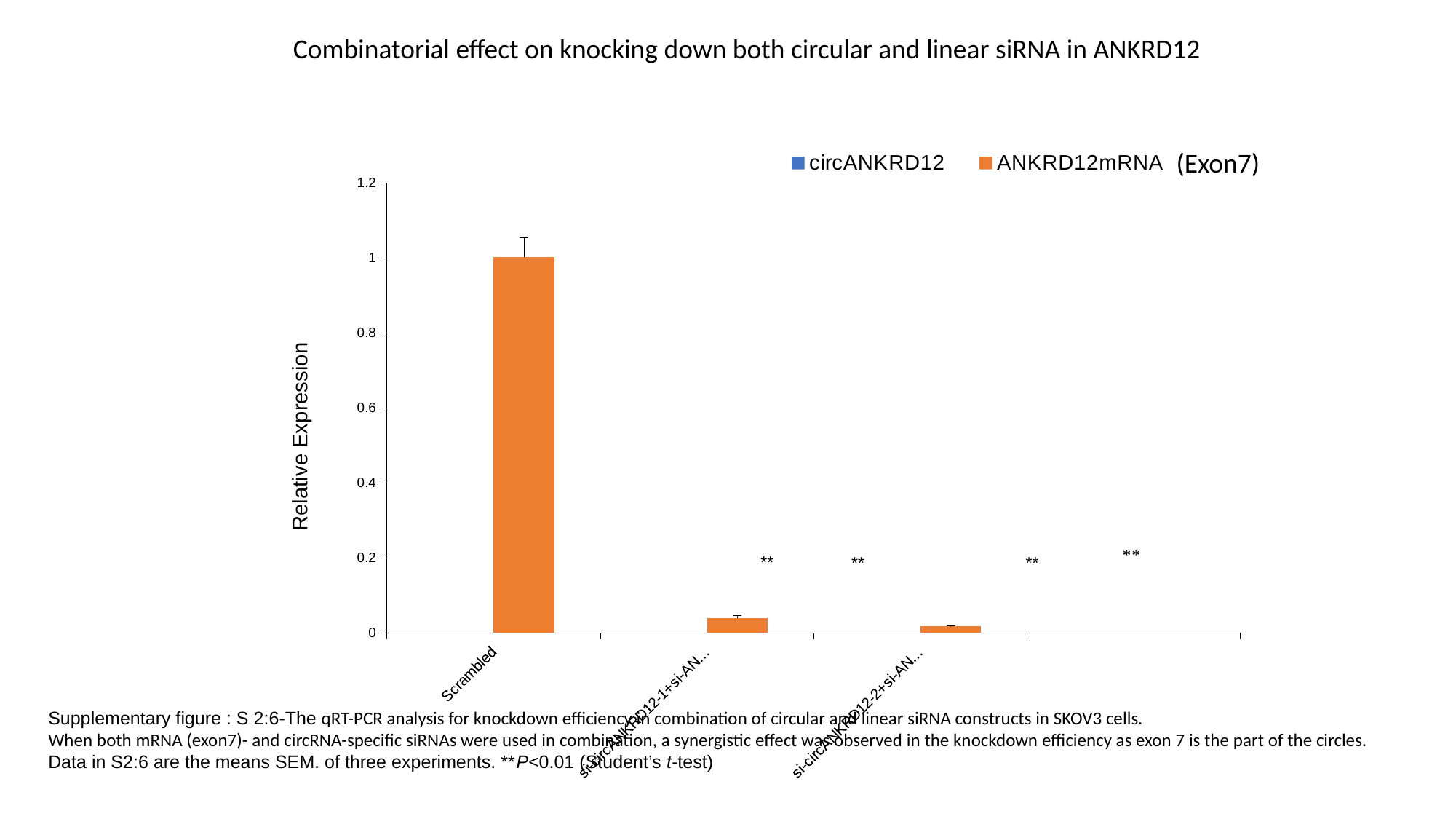

Combinatorial effect on knocking down both circular and linear siRNA in ANKRD12
### Chart
| Category | circANKRD12 | ANKRD12mRNA |
|---|---|---|
| Scrambled | 1.010701935467998 | 1.001327210916572 |
| si-circANKRD12-1+si-ANKRD12 mRNA | 0.0290625852961027 | 0.0400043159607154 |
| si-circANKRD12-2+si-ANKRD12 mRNA | 0.0122708767256109 | 0.0172923629494298 |(Exon7)
Relative Expression
**
**
**
Supplementary figure : S 2:6-The qRT-PCR analysis for knockdown efficiency in combination of circular and linear siRNA constructs in SKOV3 cells.
When both mRNA (exon7)- and circRNA-specific siRNAs were used in combination, a synergistic effect was observed in the knockdown efficiency as exon 7 is the part of the circles.
Data in S2:6 are the means SEM. of three experiments. **P<0.01 (Student’s t-test)

## Slide 7
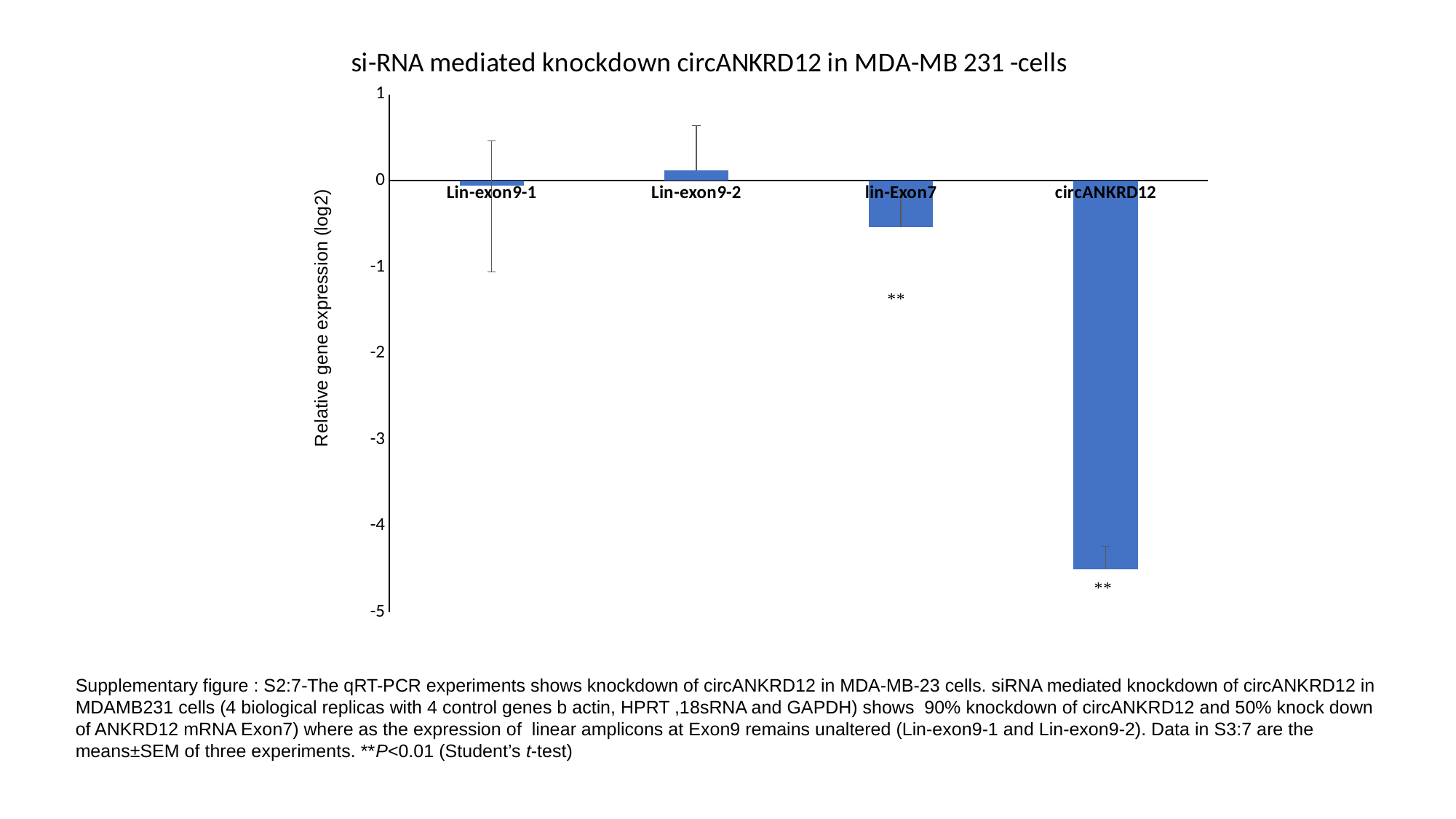

### Chart: si-RNA mediated knockdown circANKRD12 in MDA-MB 231 -cells
| Category | si-circANKRD12 |
|---|---|
| Lin-exon9-1 | -0.0577303381235485 |
| Lin-exon9-2 | 0.119735468062714 |
| lin-Exon7 | -0.540501771970829 |
| circANKRD12 | -4.503767996997483 |Supplementary figure : S2:7-The qRT-PCR experiments shows knockdown of circANKRD12 in MDA-MB-23 cells. siRNA mediated knockdown of circANKRD12 in MDAMB231 cells (4 biological replicas with 4 control genes b actin, HPRT ,18sRNA and GAPDH) shows 90% knockdown of circANKRD12 and 50% knock down of ANKRD12 mRNA Exon7) where as the expression of linear amplicons at Exon9 remains unaltered (Lin-exon9-1 and Lin-exon9-2). Data in S3:7 are the means±SEM of three experiments. **P<0.01 (Student’s t-test)

## Slide 8
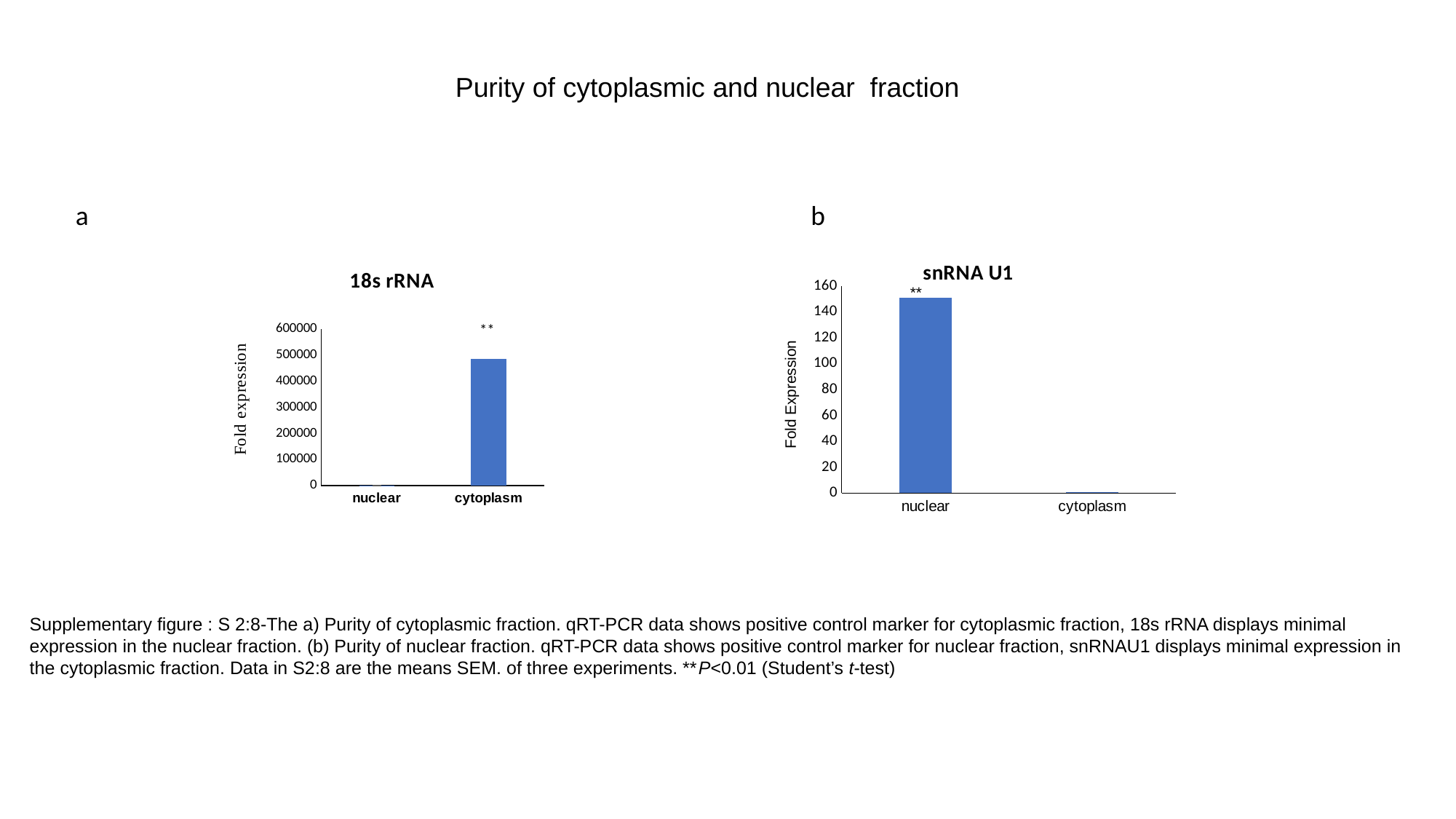

Purity of cytoplasmic and nuclear fraction
a
b
### Chart:
| Category | snRNA U1 |
|---|---|
| nuclear | 150.8116236603091 |
| cytoplasm | 1.0 |
### Chart: 18s rRNA
| Category | 18srRNA |
|---|---|
| nuclear | 1.0 |
| cytoplasm | 487103.3334571714 |**
**
Fold Expression
Supplementary figure : S 2:8-The a) Purity of cytoplasmic fraction. qRT-PCR data shows positive control marker for cytoplasmic fraction, 18s rRNA displays minimal expression in the nuclear fraction. (b) Purity of nuclear fraction. qRT-PCR data shows positive control marker for nuclear fraction, snRNAU1 displays minimal expression in the cytoplasmic fraction. Data in S2:8 are the means SEM. of three experiments. **P<0.01 (Student’s t-test)

## Slide 9
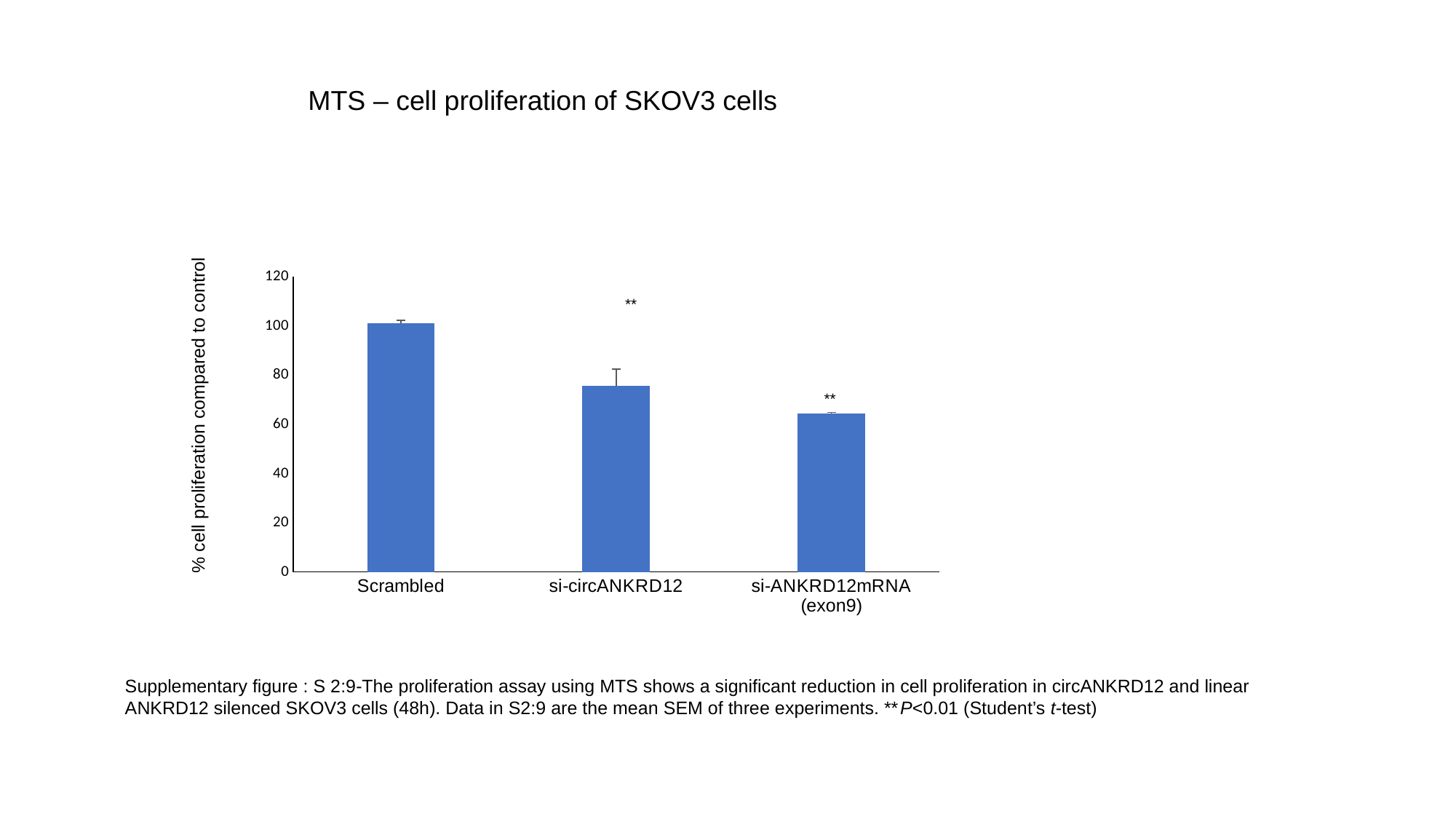

MTS – cell proliferation of SKOV3 cells
### Chart
| Category | |
|---|---|
| Scrambled | 101.1298076923077 |
| si-circANKRD12 | 75.57891058060598 |
| si-ANKRD12mRNA (exon9) | 64.32210484278326 |**
**
% cell proliferation compared to control
Supplementary figure : S 2:9-The proliferation assay using MTS shows a significant reduction in cell proliferation in circANKRD12 and linear ANKRD12 silenced SKOV3 cells (48h). Data in S2:9 are the mean SEM of three experiments. **P<0.01 (Student’s t-test)

## Slide 10
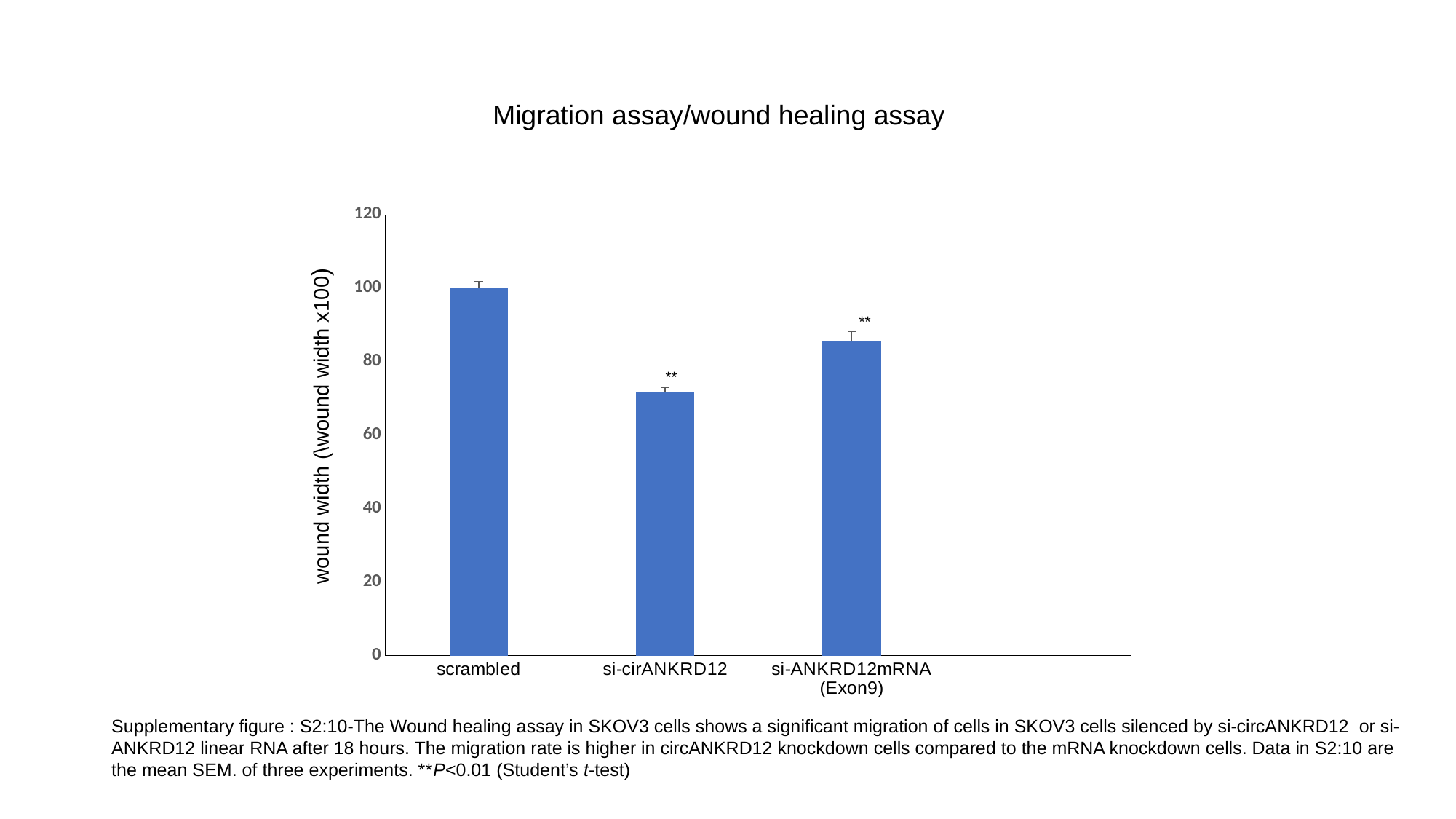

Migration assay/wound healing assay
### Chart
| Category | |
|---|---|
| scrambled | 100.1812309174052 |
| si-cirANKRD12 | 71.72799571508965 |
| si-ANKRD12mRNA (Exon9) | 85.37794454191408 |**
**
wound width (\wound width x100)
Supplementary figure : S2:10-The Wound healing assay in SKOV3 cells shows a significant migration of cells in SKOV3 cells silenced by si-circANKRD12 or si-ANKRD12 linear RNA after 18 hours. The migration rate is higher in circANKRD12 knockdown cells compared to the mRNA knockdown cells. Data in S2:10 are the mean SEM. of three experiments. **P<0.01 (Student’s t-test)

## Slide 11
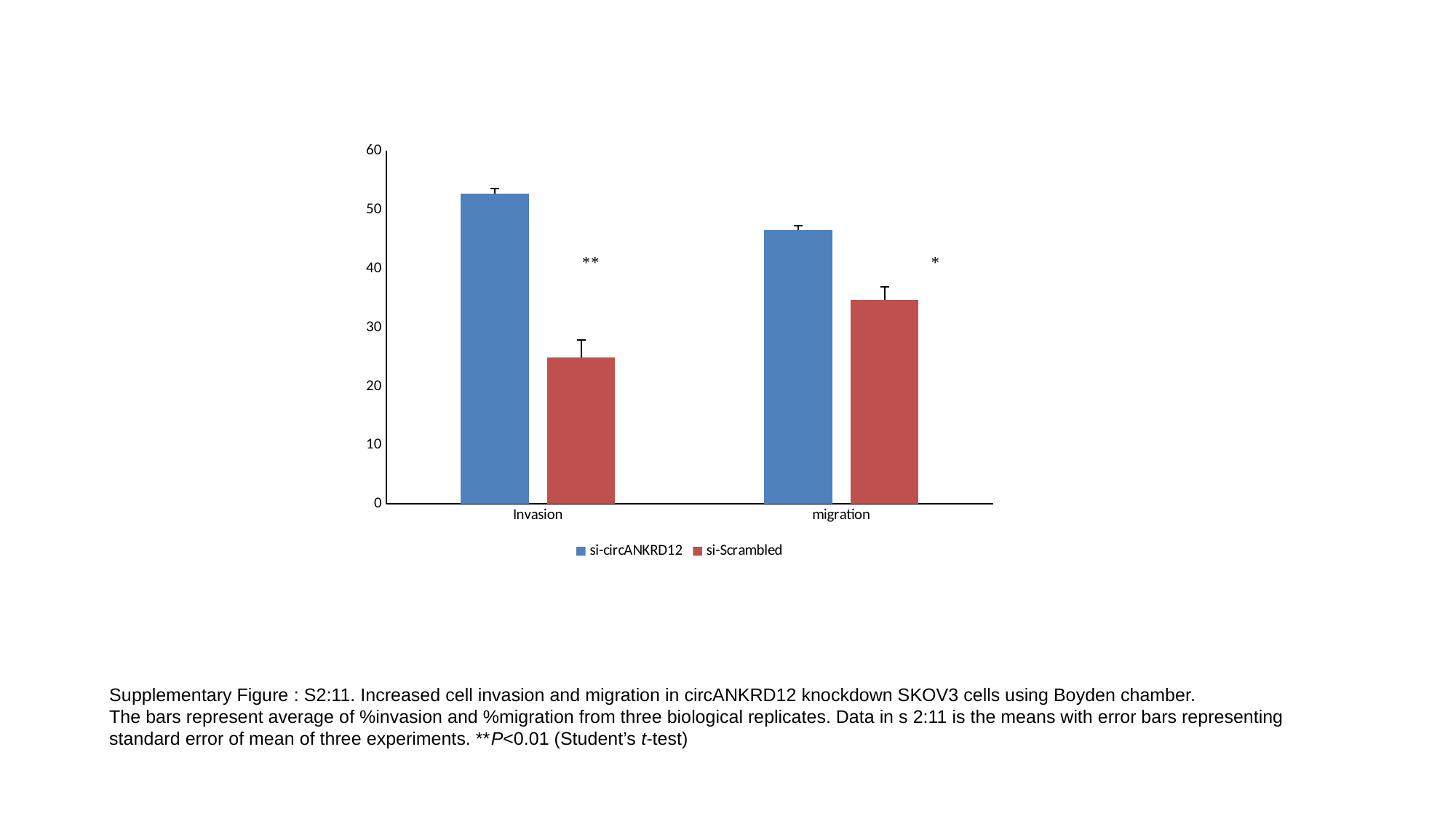

### Chart
| Category | si-circANKRD12 | si-Scrambled |
|---|---|---|
| Invasion | 52.7097003269008 | 24.95224922611076 |
| migration | 46.60891371252706 | 34.72499466187367 |Supplementary Figure : S2:11. Increased cell invasion and migration in circANKRD12 knockdown SKOV3 cells using Boyden chamber.
The bars represent average of %invasion and %migration from three biological replicates. Data in s 2:11 is the means with error bars representing
standard error of mean of three experiments. **P<0.01 (Student’s t-test)

## Slide 12
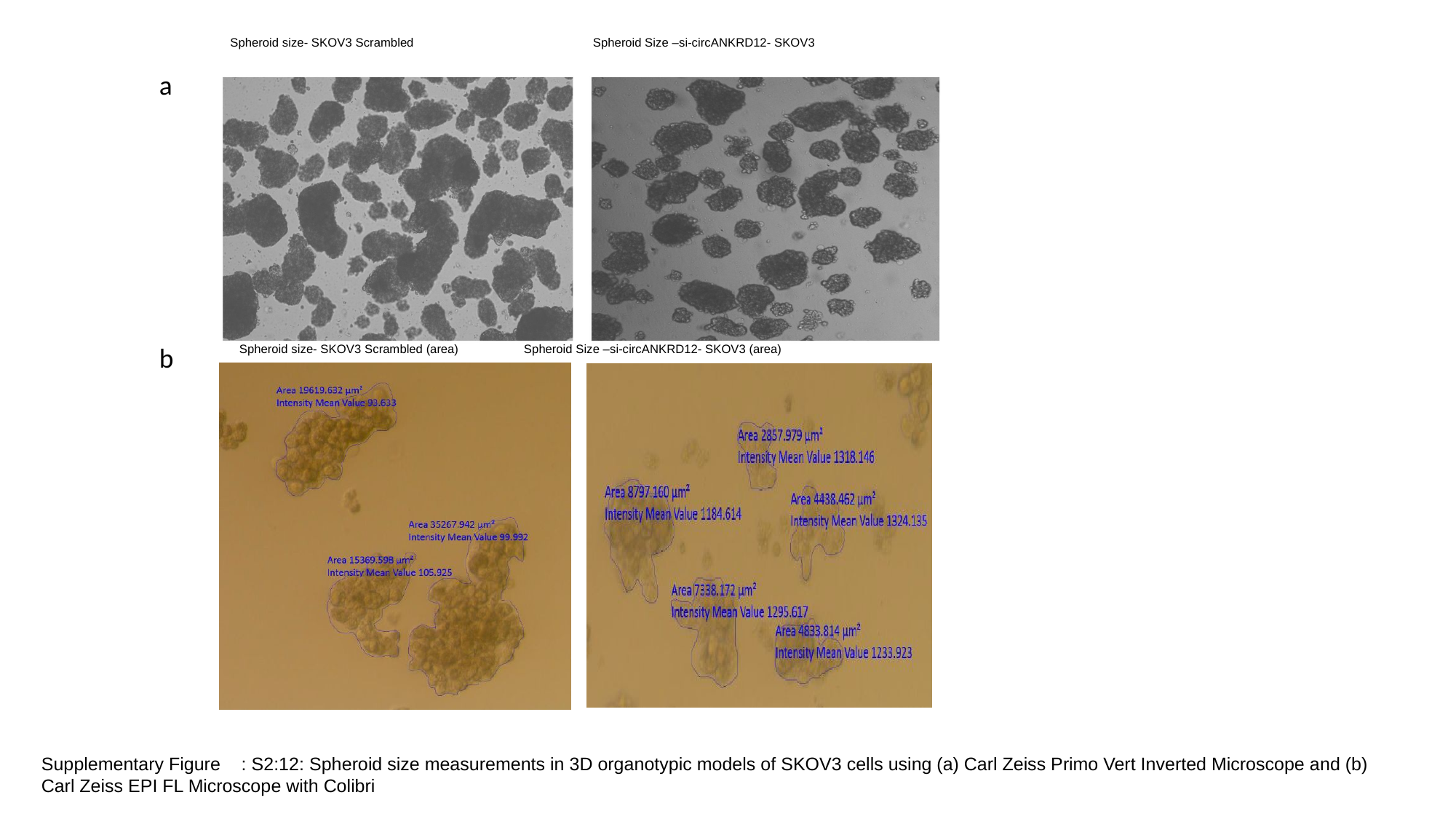

Spheroid size- SKOV3 Scrambled Spheroid Size –si-circANKRD12- SKOV3
a
b
Spheroid size- SKOV3 Scrambled (area) Spheroid Size –si-circANKRD12- SKOV3 (area)
Supplementary Figure : S2:12: Spheroid size measurements in 3D organotypic models of SKOV3 cells using (a) Carl Zeiss Primo Vert Inverted Microscope and (b) Carl Zeiss EPI FL Microscope with Colibri

## Slide 13
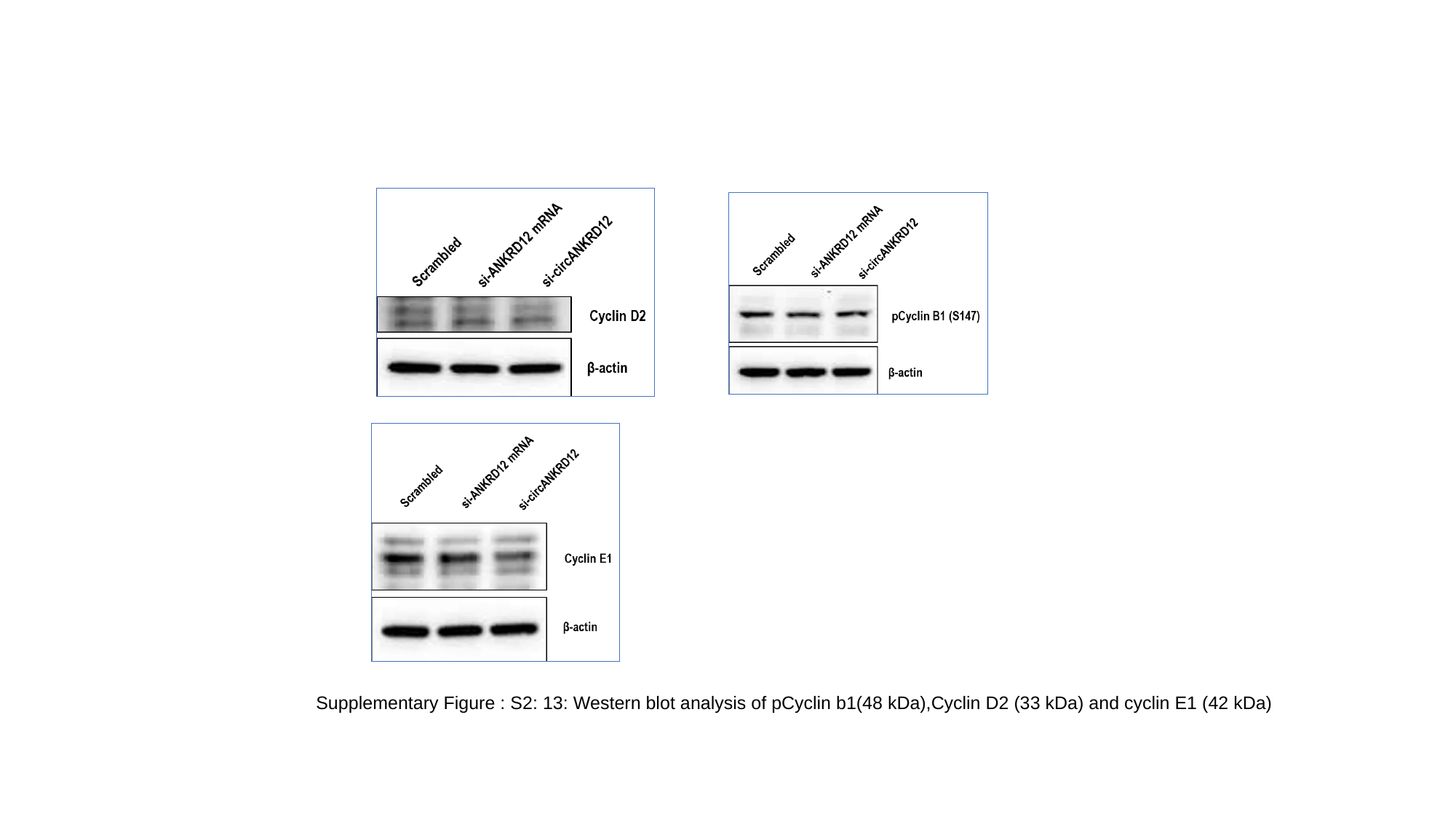

Supplementary Figure : S2: 13: Western blot analysis of pCyclin b1(48 kDa),Cyclin D2 (33 kDa) and cyclin E1 (42 kDa)

## Slide 14
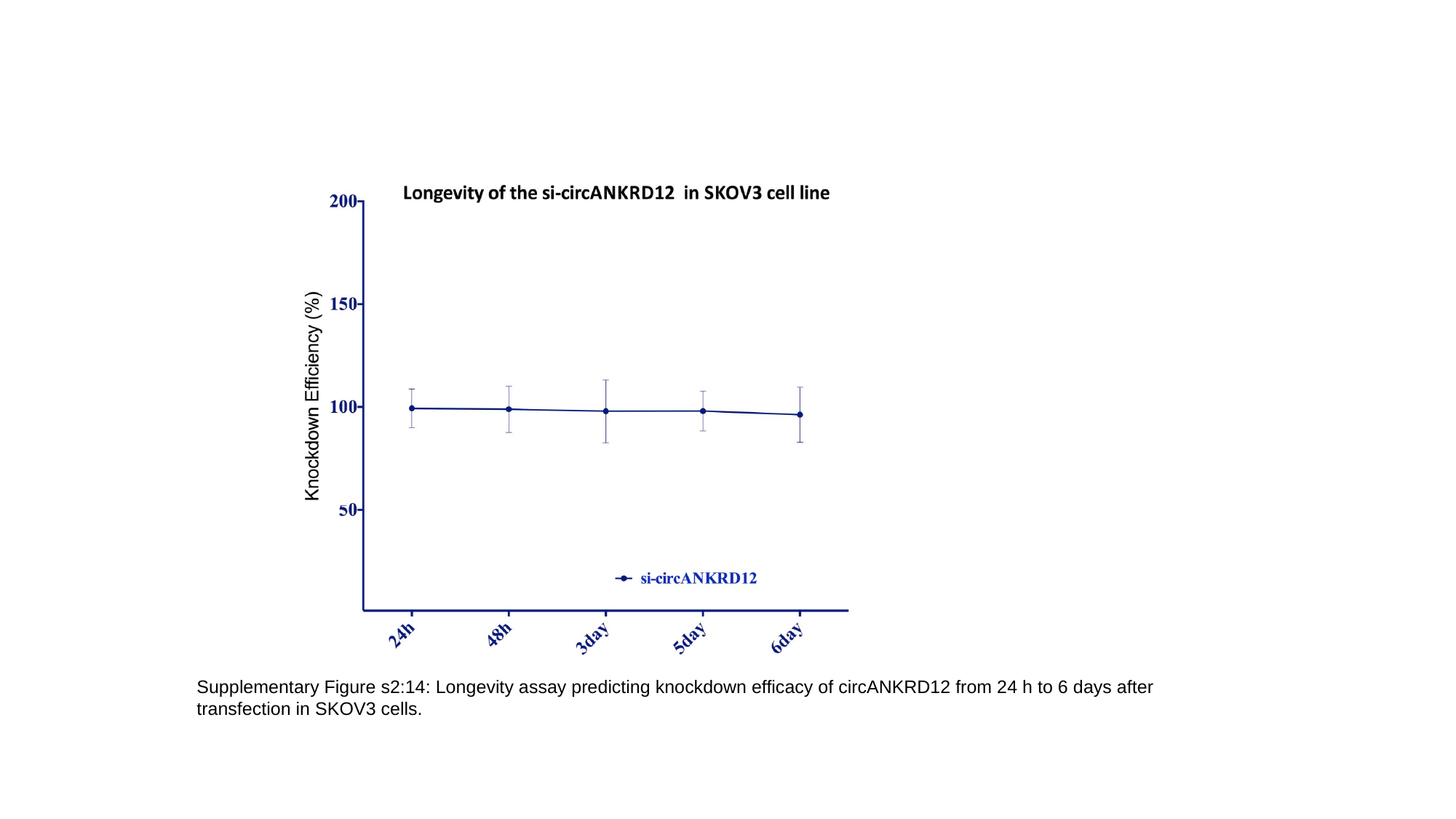

Supplementary Figure s2:14: Longevity assay predicting knockdown efficacy of circANKRD12 from 24 h to 6 days after transfection in SKOV3 cells.

## Slide 15
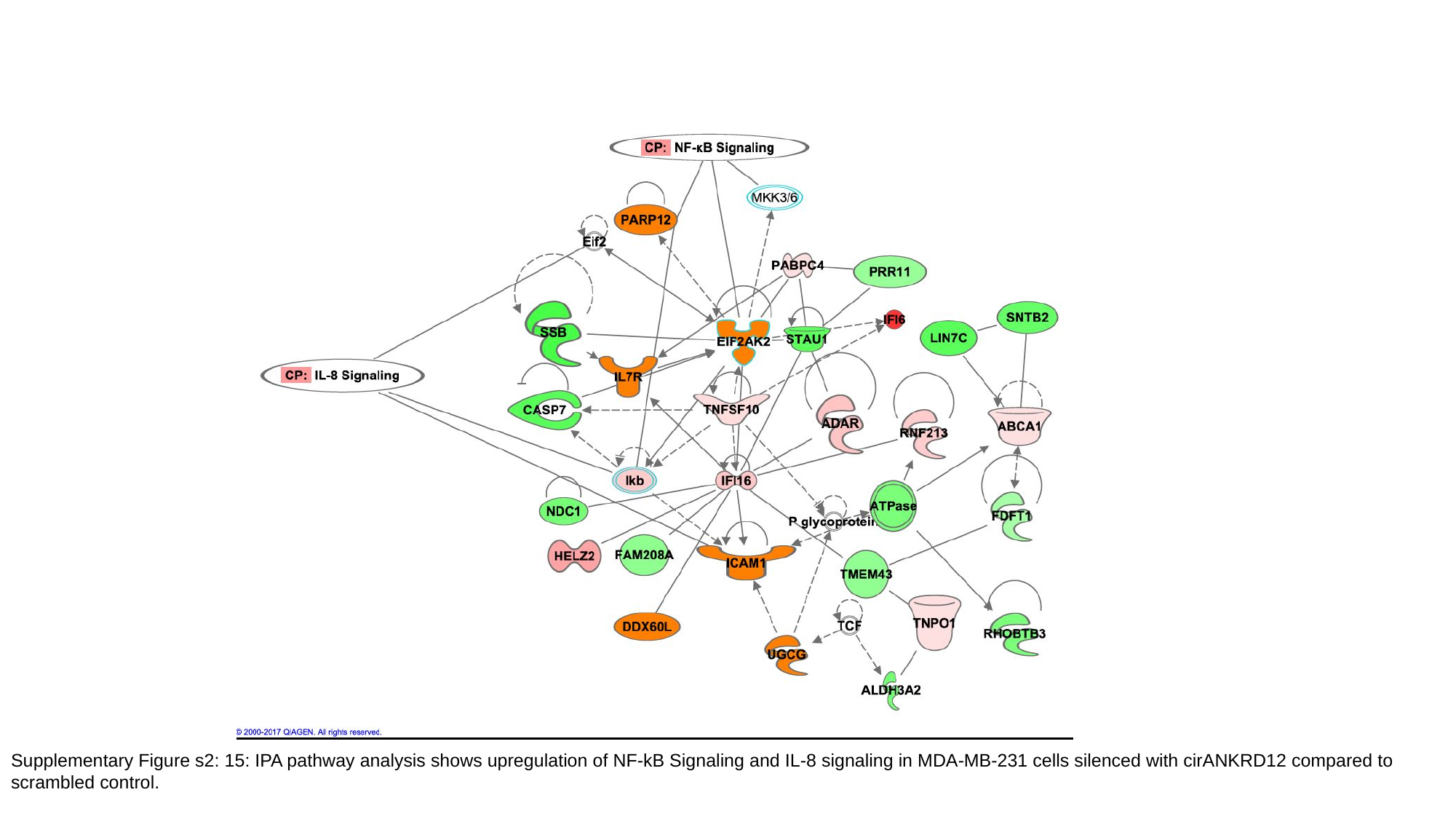

Supplementary Figure s2: 15: IPA pathway analysis shows upregulation of NF-kB Signaling and IL-8 signaling in MDA-MB-231 cells silenced with cirANKRD12 compared to scrambled control.

## Slide 16
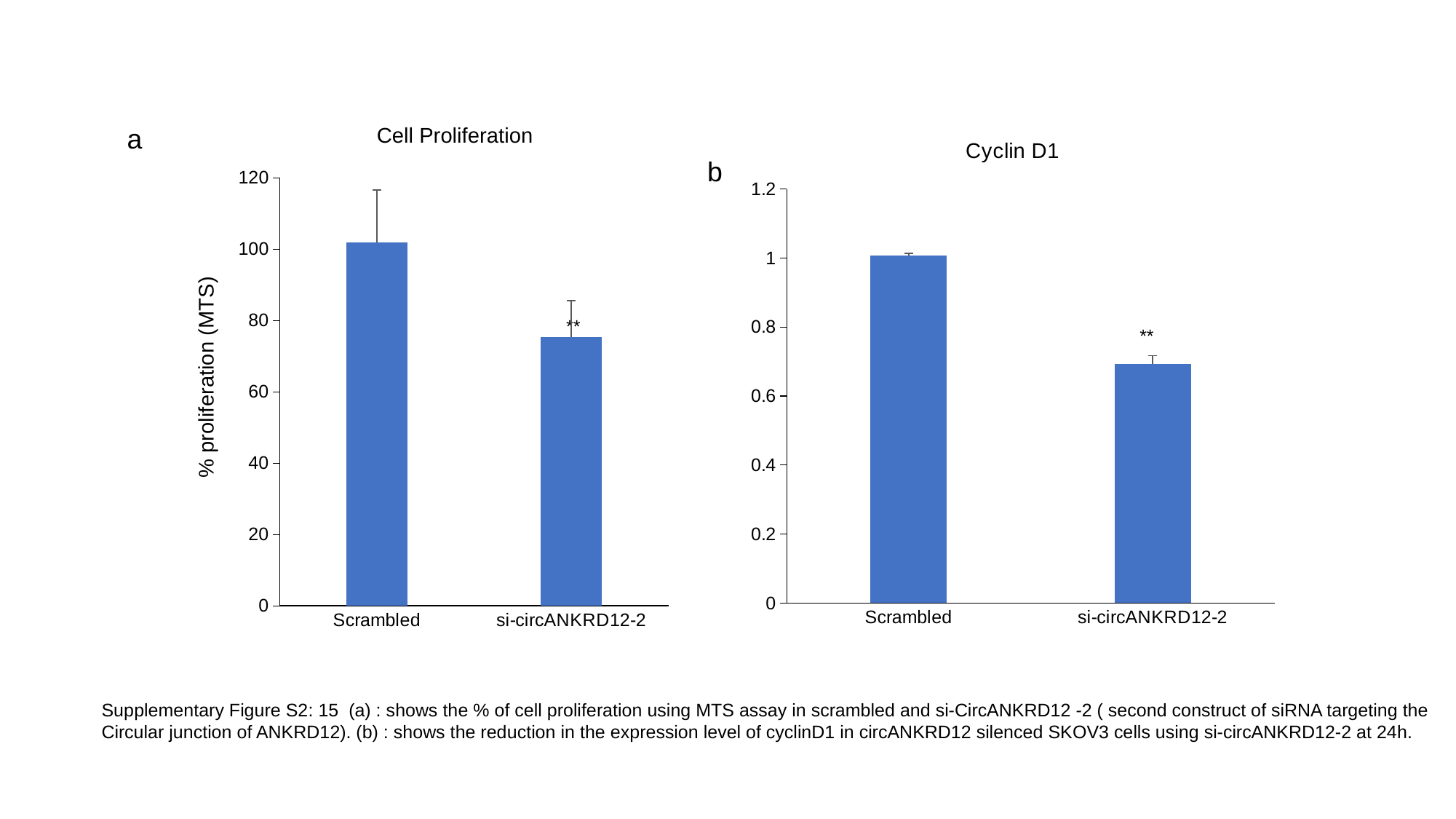

a
Cell Proliferation
### Chart:
| Category | Cyclin D1 |
|---|---|
| Scrambled | 1.0079521710713457 |
| si-circANKRD12-2 | 0.6922636537277057 |b
### Chart
| Category | |
|---|---|
| Scrambled | 101.90176048685066 |
| si-circANKRD12-2 | 75.38973498021349 |**
**
% proliferation (MTS)
Supplementary Figure S2: 15 (a) : shows the % of cell proliferation using MTS assay in scrambled and si-CircANKRD12 -2 ( second construct of siRNA targeting the
Circular junction of ANKRD12). (b) : shows the reduction in the expression level of cyclinD1 in circANKRD12 silenced SKOV3 cells using si-circANKRD12-2 at 24h.
