# Supplementary material for: Silencing of ANKRD12 circRNA induces molecular and functional changes associated with invasive phenotypes
Source: BMC Cancer. 2019 Jun 11;19:565. doi: 10.1186/s12885-019-5723-0 (PMC6558796; doi:10.1186/s12885-019-5723-0)
Supplement: Supplementary file 4 — Tables S1-S5. Tables representing primers used for different gene expression studies, siRNAs and pathways involved in circANKRD12 gene knockdown condition. (PPTX 3253 kb) (PPTX 3268 kb) [file 12885_2019_5723_MOESM4_ESM.pptx]

## Slide 1
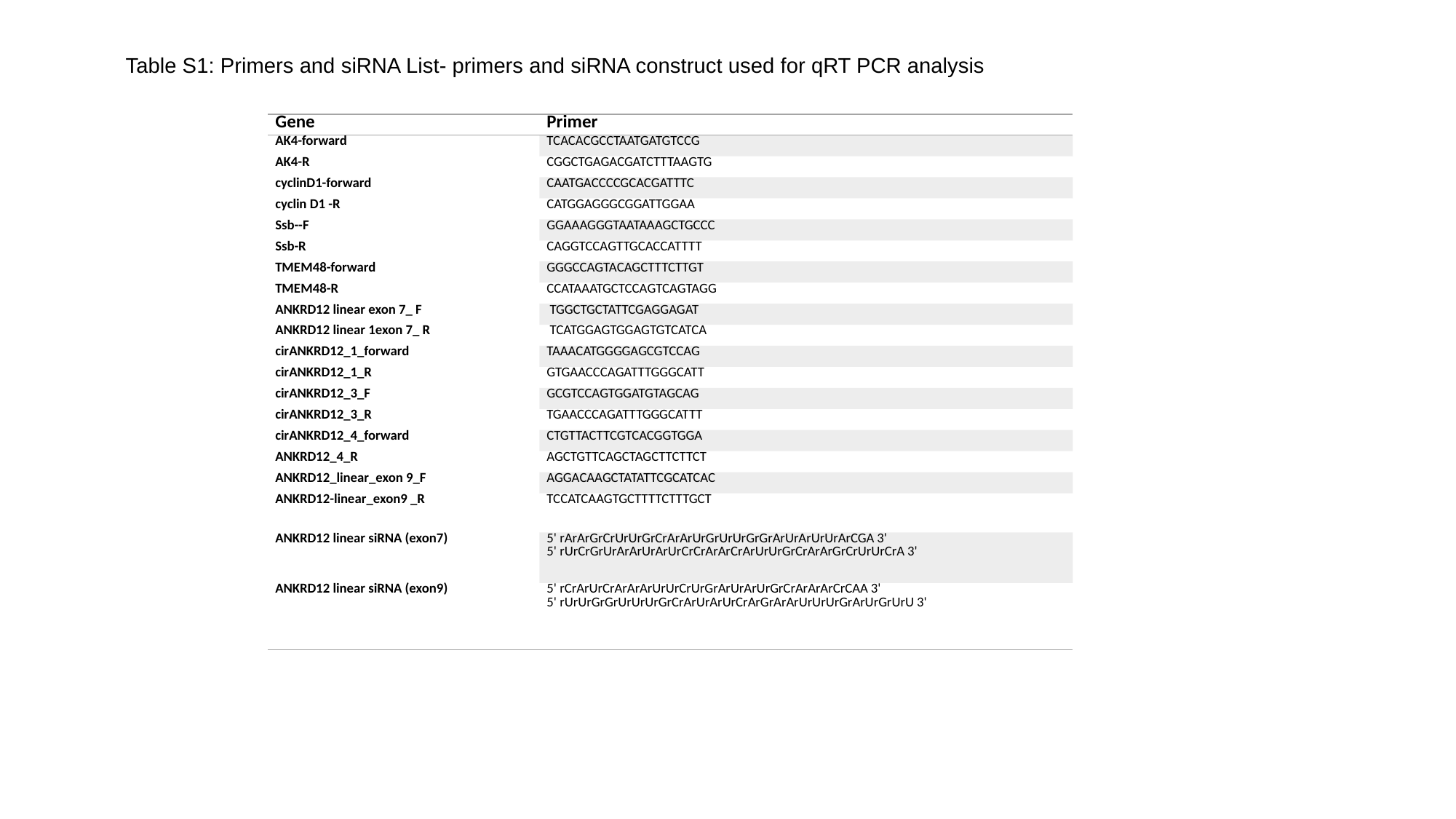

Table S1: Primers and siRNA List- primers and siRNA construct used for qRT PCR analysis
| Gene | Primer |
| --- | --- |
| AK4-forward | TCACACGCCTAATGATGTCCG |
| AK4-R | CGGCTGAGACGATCTTTAAGTG |
| cyclinD1-forward | CAATGACCCCGCACGATTTC |
| cyclin D1 -R | CATGGAGGGCGGATTGGAA |
| Ssb--F | GGAAAGGGTAATAAAGCTGCCC |
| Ssb-R | CAGGTCCAGTTGCACCATTTT |
| TMEM48-forward | GGGCCAGTACAGCTTTCTTGT |
| TMEM48-R | CCATAAATGCTCCAGTCAGTAGG |
| ANKRD12 linear exon 7\_ F | TGGCTGCTATTCGAGGAGAT |
| ANKRD12 linear 1exon 7\_ R | TCATGGAGTGGAGTGTCATCA |
| cirANKRD12\_1\_forward | TAAACATGGGGAGCGTCCAG |
| cirANKRD12\_1\_R | GTGAACCCAGATTTGGGCATT |
| cirANKRD12\_3\_F | GCGTCCAGTGGATGTAGCAG |
| cirANKRD12\_3\_R | TGAACCCAGATTTGGGCATTT |
| cirANKRD12\_4\_forward | CTGTTACTTCGTCACGGTGGA |
| ANKRD12\_4\_R | AGCTGTTCAGCTAGCTTCTTCT |
| ANKRD12\_linear\_exon 9\_F | AGGACAAGCTATATTCGCATCAC |
| ANKRD12-linear\_exon9 \_R | TCCATCAAGTGCTTTTCTTTGCT |
| ANKRD12 linear siRNA (exon7) | 5' rArArGrCrUrUrGrCrArArUrGrUrUrGrGrArUrArUrUrArCGA 3' 5' rUrCrGrUrArArUrArUrCrCrArArCrArUrUrGrCrArArGrCrUrUrCrA 3' |
| ANKRD12 linear siRNA (exon9) | 5' rCrArUrCrArArArUrUrCrUrGrArUrArUrGrCrArArArCrCAA 3' 5' rUrUrGrGrUrUrUrGrCrArUrArUrCrArGrArArUrUrUrGrArUrGrUrU 3' |

## Slide 2
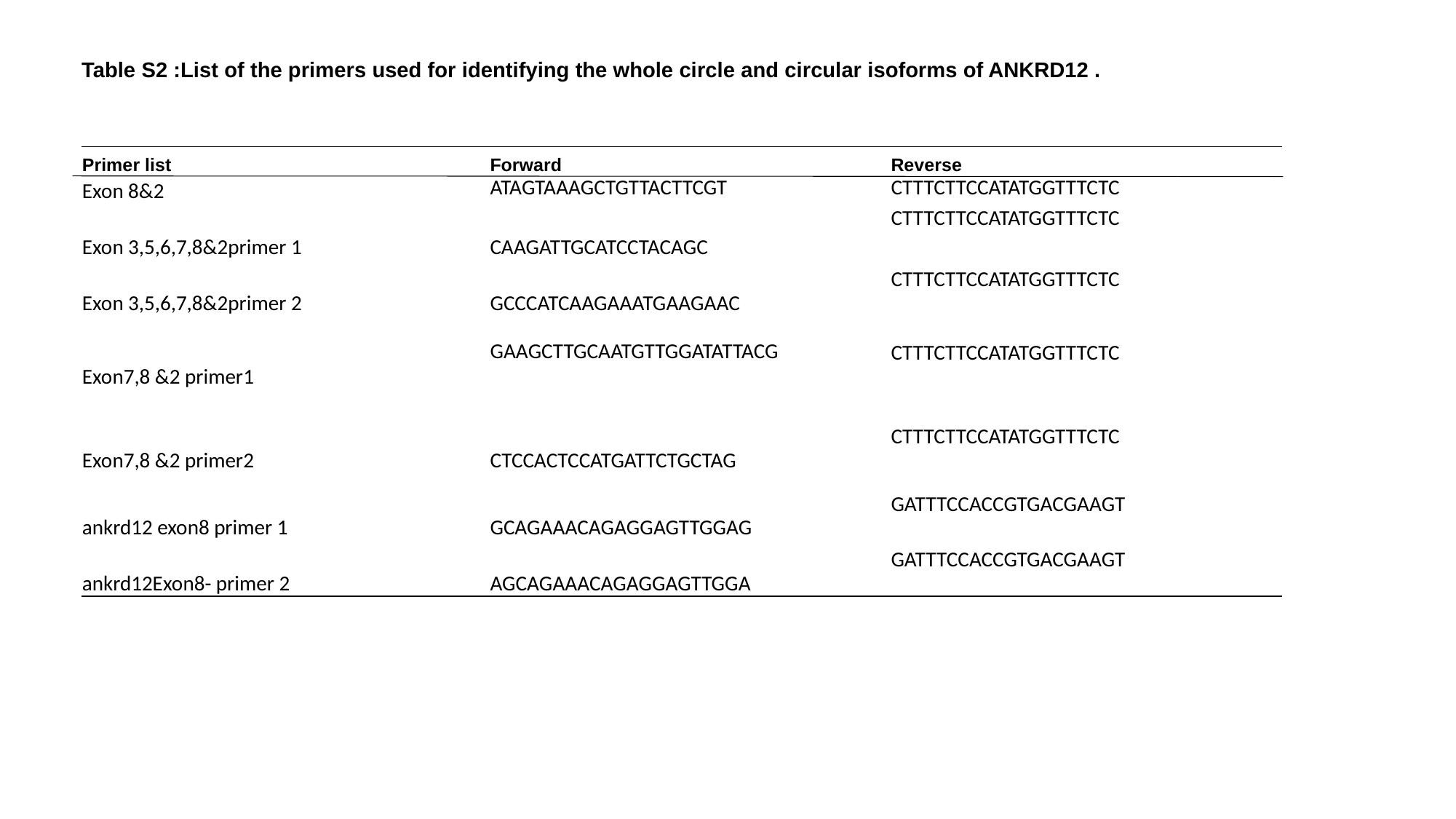

Table S2 :List of the primers used for identifying the whole circle and circular isoforms of ANKRD12 .
| Primer list | Forward | Reverse |
| --- | --- | --- |
| Exon 8&2 | ATAGTAAAGCTGTTACTTCGT | CTTTCTTCCATATGGTTTCTC |
| Exon 3,5,6,7,8&2primer 1 | CAAGATTGCATCCTACAGC | CTTTCTTCCATATGGTTTCTC |
| Exon 3,5,6,7,8&2primer 2 | GCCCATCAAGAAATGAAGAAC | CTTTCTTCCATATGGTTTCTC |
| Exon7,8 &2 primer1 | GAAGCTTGCAATGTTGGATATTACG | CTTTCTTCCATATGGTTTCTC |
| Exon7,8 &2 primer2 | CTCCACTCCATGATTCTGCTAG | CTTTCTTCCATATGGTTTCTC |
| ankrd12 exon8 primer 1 | GCAGAAACAGAGGAGTTGGAG | GATTTCCACCGTGACGAAGT |
| ankrd12Exon8- primer 2 | AGCAGAAACAGAGGAGTTGGA | GATTTCCACCGTGACGAAGT |

## Slide 3
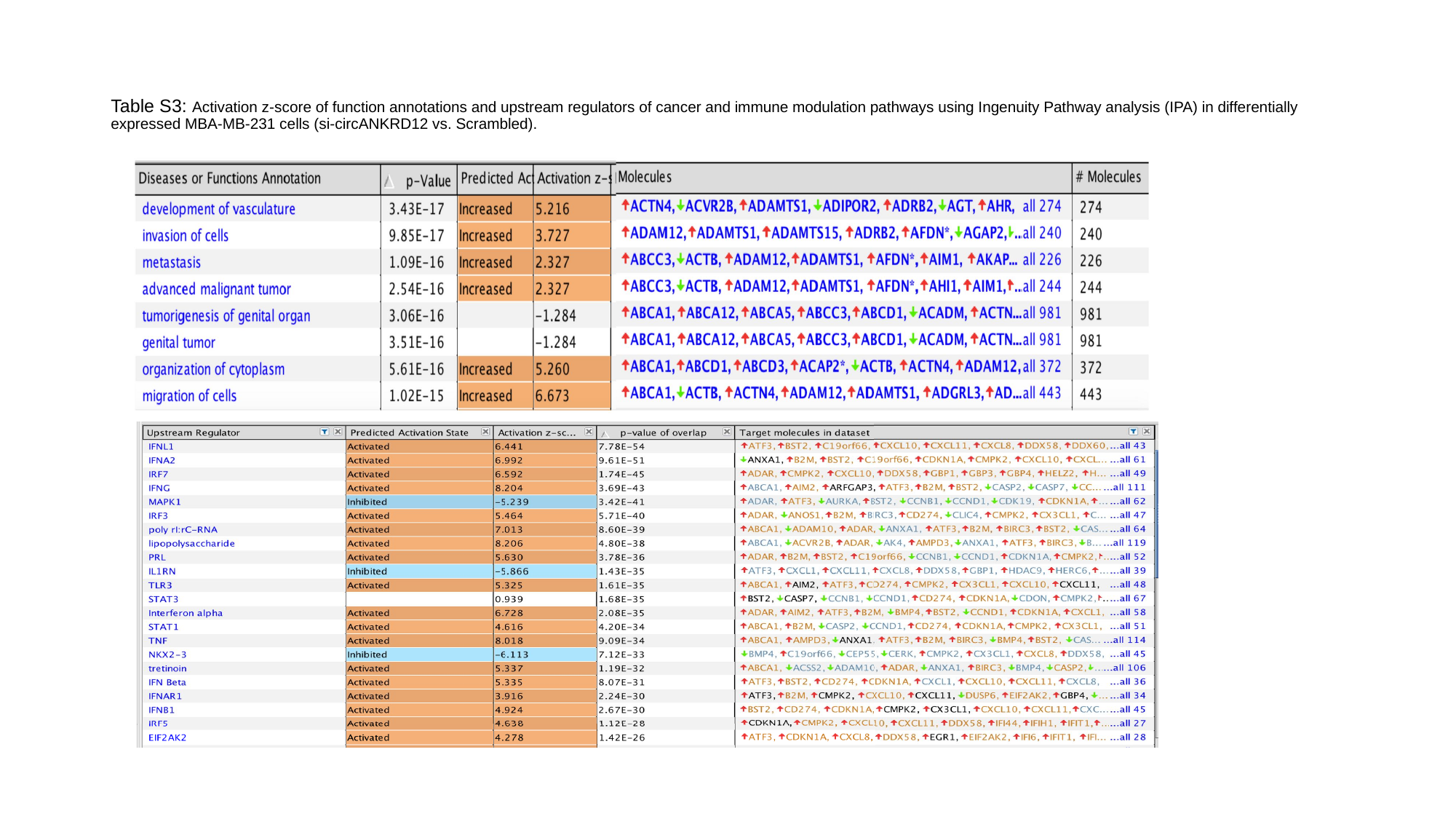

# Table S3: Activation z-score of function annotations and upstream regulators of cancer and immune modulation pathways using Ingenuity Pathway analysis (IPA) in differentially expressed MBA-MB-231 cells (si-circANKRD12 vs. Scrambled).

## Slide 4
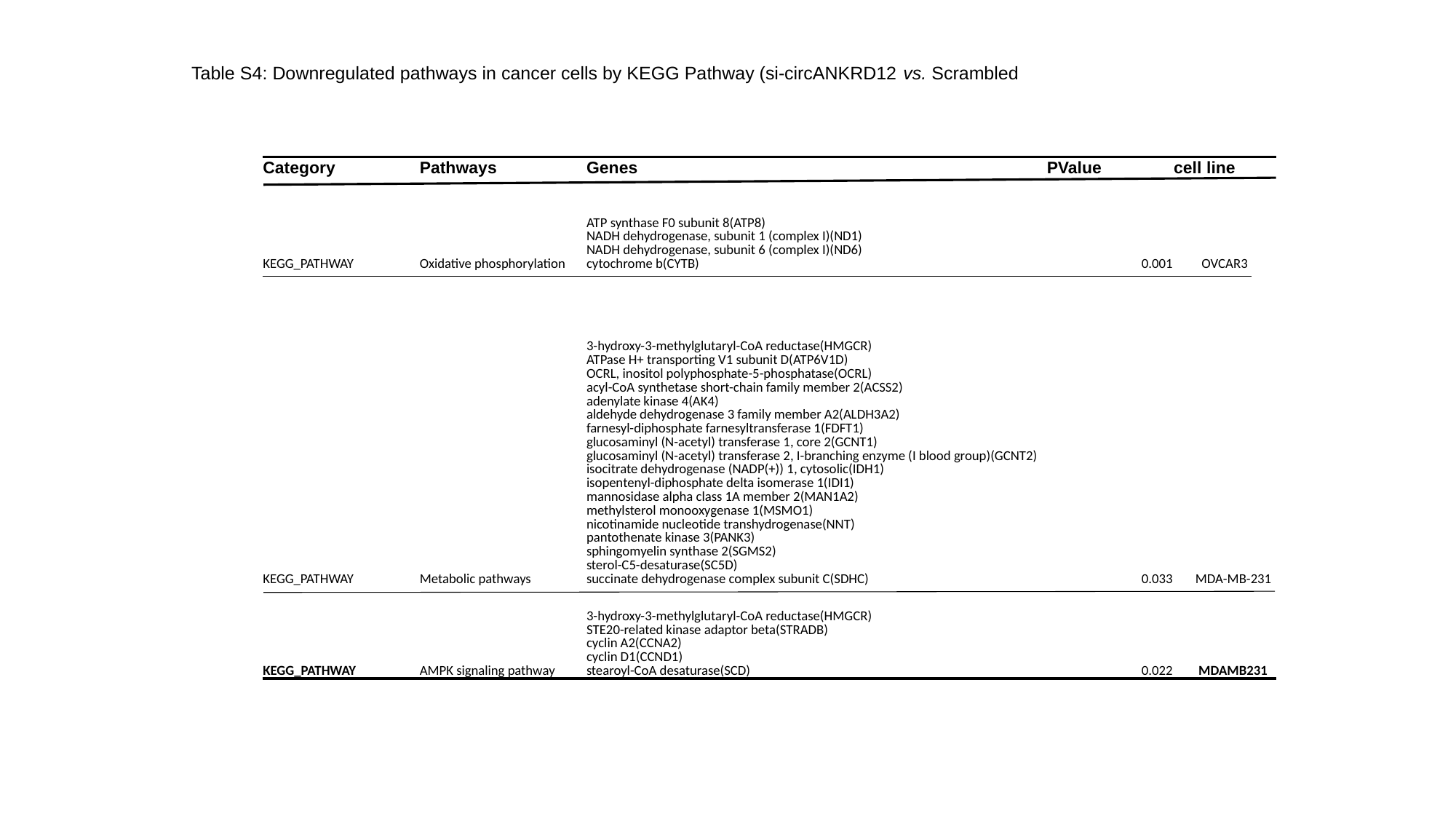

Table S4: Downregulated pathways in cancer cells by KEGG Pathway (si-circANKRD12 vs. Scrambled
| Category | Pathways | Genes | PValue | cell line |
| --- | --- | --- | --- | --- |
| KEGG\_PATHWAY | Oxidative phosphorylation | ATP synthase F0 subunit 8(ATP8) NADH dehydrogenase, subunit 1 (complex I)(ND1) NADH dehydrogenase, subunit 6 (complex I)(ND6) cytochrome b(CYTB) | 0.001 | OVCAR3 |
| KEGG\_PATHWAY | Metabolic pathways | 3-hydroxy-3-methylglutaryl-CoA reductase(HMGCR) ATPase H+ transporting V1 subunit D(ATP6V1D) OCRL, inositol polyphosphate-5-phosphatase(OCRL) acyl-CoA synthetase short-chain family member 2(ACSS2) adenylate kinase 4(AK4) aldehyde dehydrogenase 3 family member A2(ALDH3A2) farnesyl-diphosphate farnesyltransferase 1(FDFT1) glucosaminyl (N-acetyl) transferase 1, core 2(GCNT1) glucosaminyl (N-acetyl) transferase 2, I-branching enzyme (I blood group)(GCNT2) isocitrate dehydrogenase (NADP(+)) 1, cytosolic(IDH1) isopentenyl-diphosphate delta isomerase 1(IDI1) mannosidase alpha class 1A member 2(MAN1A2) methylsterol monooxygenase 1(MSMO1) nicotinamide nucleotide transhydrogenase(NNT) pantothenate kinase 3(PANK3) sphingomyelin synthase 2(SGMS2) sterol-C5-desaturase(SC5D) succinate dehydrogenase complex subunit C(SDHC) | 0.033 | MDA-MB-231 |
| KEGG\_PATHWAY | AMPK signaling pathway | 3-hydroxy-3-methylglutaryl-CoA reductase(HMGCR) STE20-related kinase adaptor beta(STRADB) cyclin A2(CCNA2) cyclin D1(CCND1) stearoyl-CoA desaturase(SCD) | 0.022 | MDAMB231 |

## Slide 5
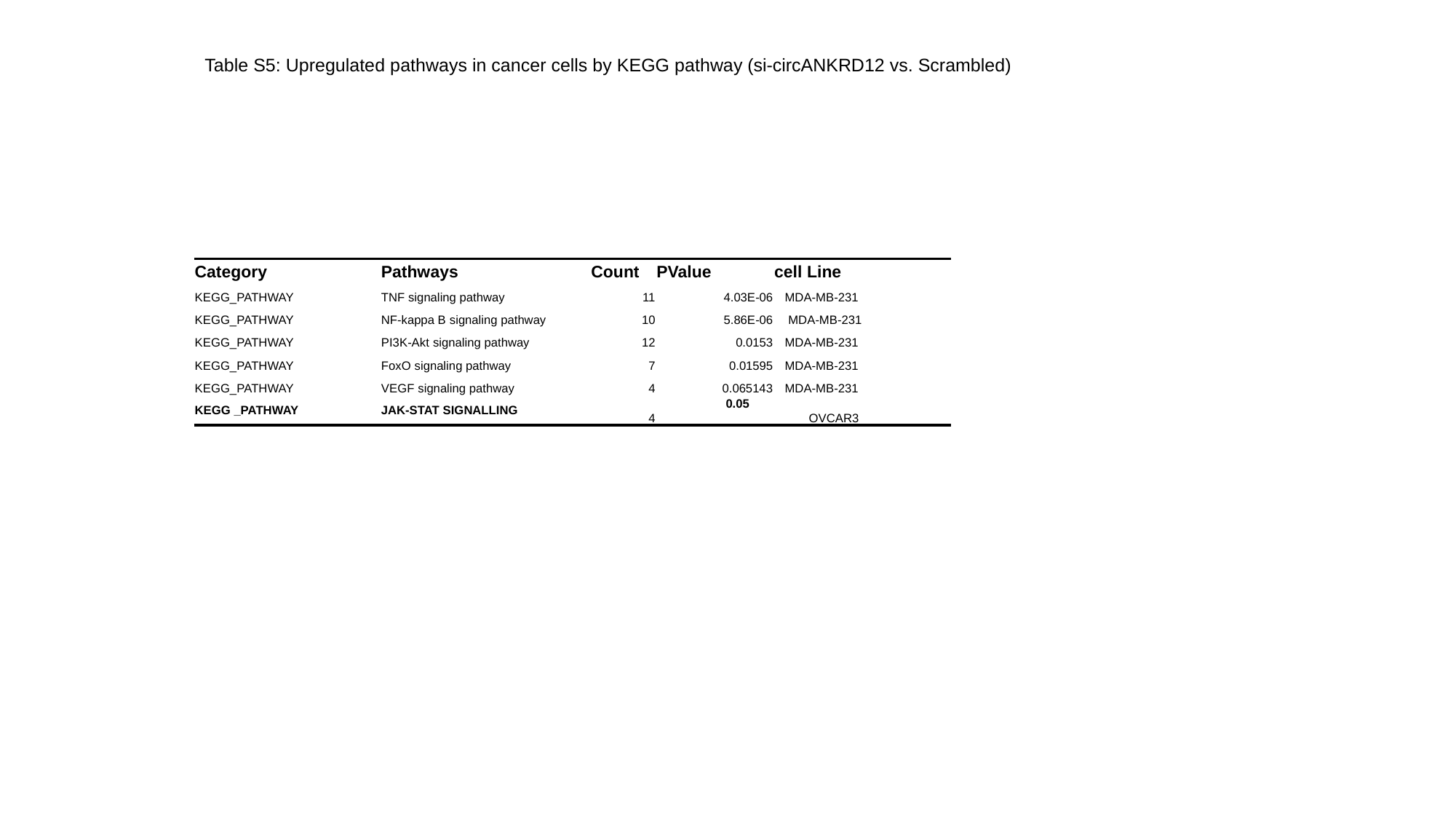

Table S5: Upregulated pathways in cancer cells by KEGG pathway (si-circANKRD12 vs. Scrambled)
| Category | Pathways | Count | PValue | cell Line |
| --- | --- | --- | --- | --- |
| KEGG\_PATHWAY | TNF signaling pathway | 11 | 4.03E-06 | MDA-MB-231 |
| KEGG\_PATHWAY | NF-kappa B signaling pathway | 10 | 5.86E-06 | MDA-MB-231 |
| KEGG\_PATHWAY | PI3K-Akt signaling pathway | 12 | 0.0153 | MDA-MB-231 |
| KEGG\_PATHWAY | FoxO signaling pathway | 7 | 0.01595 | MDA-MB-231 |
| KEGG\_PATHWAY | VEGF signaling pathway | 4 | 0.065143 | MDA-MB-231 |
| KEGG \_PATHWAY JAK-STAT SIGNALLING | | 4 | 0.05 | OVCAR3 |
